# Glycation of α-synuclein hampers its binding to synaptic-like vesicles and its driving effect on their fusion

**DOI:** 10.1007/s00018-022-04373-4

**Published:** 2022-06-04

**Authors:** Ana Belén Uceda, Juan Frau, Bartolomé Vilanova, Miquel Adrover

**Affiliations:** Departament de Química, Institut Universitari d’Investigació en Ciències de la Salut (IUNICS), Institut de Recerca en Ciències de la Salut (IdISBa), Universitat de les Illes Balears, Ed. Mateu Orfila i Rotger, Ctra. Valldemossa km 7.5, 07122 Palma, Spain

**Keywords:** Human α-synuclein, Synaptic vesicles, Glycation, Protein structure

## Abstract

Parkinson’s disease (PD) is one of the most prevalent neurodegenerative disorders affecting the worldwide population. One of its hallmarks is the intraneuronal accumulation of insoluble Lewy bodies (LBs), which cause the death of dopaminergic neurons. α-Synuclein (αS) is the main component of these LBs and in them, it commonly contains non-enzymatic post-translational modifications, such as those resulting from its reaction with reactive carbonyl species arising as side products of the intraneuronal glycolysis (mainly methylglyoxal). Consequently, lysines of the αS found in LBs of diabetic individuals are usually carboxyethylated. A precise comprehension of the effect of N^ε^-(carboxyethyl)lysine (CEL) on the aggregation of αS and on its physiological function becomes crucial to fully understand the molecular mechanisms underlying the development of diabetes-induced PD. Consequently, we have here used a synthetic αS where all its Lys have been replaced by CEL moieties (αS-CEL), and we have studied how these modifications could impact on the neurotransmission mechanism. This study allows us to describe how the non-enzymatic glycosylation (glycation) affects the function of a protein like αS, involved in the pathogenesis of PD. CEL decreases the ability of αS to bind micelles, although the micelle-bound fraction of αS-CEL still displays an α-helical fold resembling that of the lipid-bound αS. However, CEL completely abolishes the affinity of αS towards synaptic-like vesicles and, consequently, it hampers its physiological function as a catalyst of the clustering and the fusion of the synaptic vesicles.

**Supplementary Information:**

The online version contains supplementary material available at 10.1007/s00018-022-04373-4.

## Introduction

**Fig. 1:**
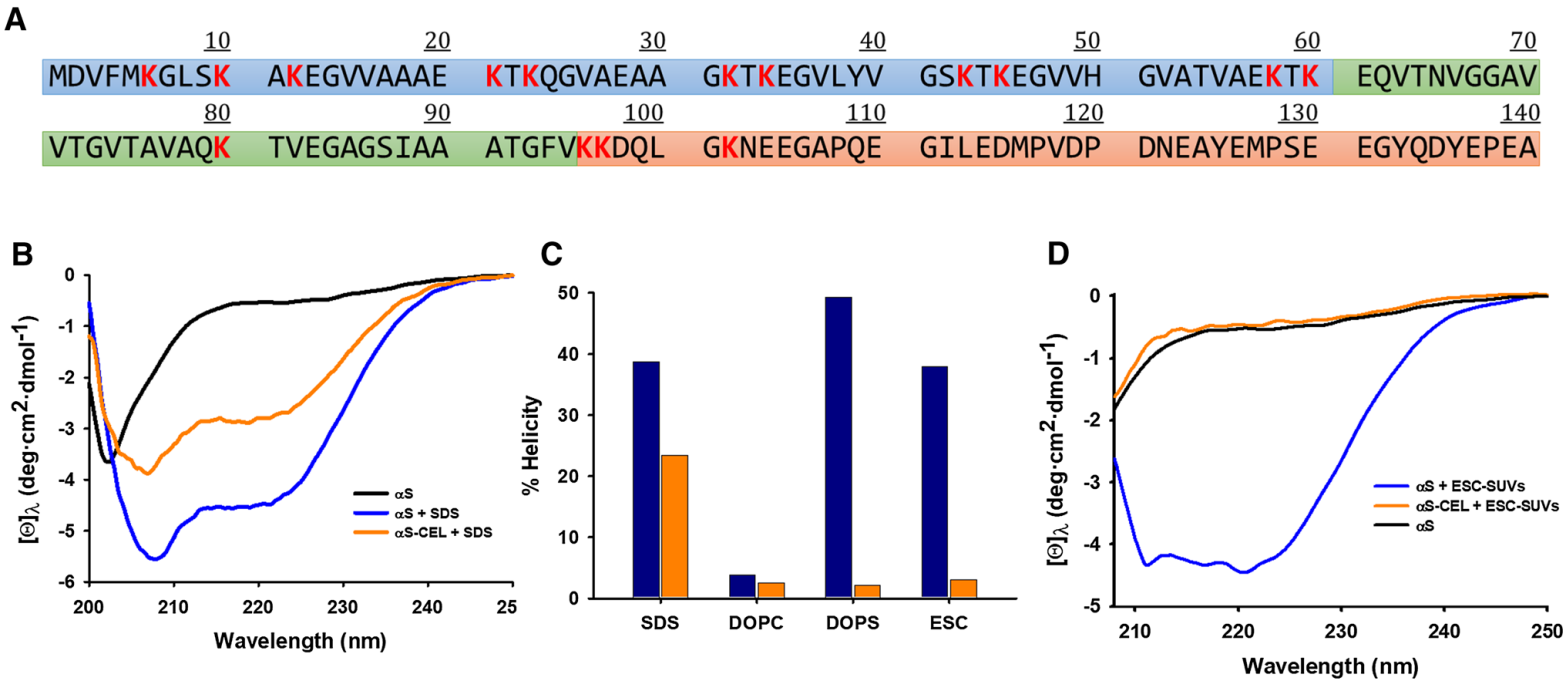
Effect of CEL formation on the α-helical folding of αS bound to SDS micelles and ESC SUVs. **A** Amino-acid sequence of αS with the regions corresponding to the different domains squared in different colours (i.e. the N-terminal domain in blue; the NAC domain in green; and the C-terminal domain in orange) and the Lys residues coloured in red. **B** Overlapping of the far-UV CD spectra of αS (20 µM) in the absence (black) and in the presence (blue) of SDS (10 mM) on that corresponding to αS-CEL (20 µM) in the presence of SDS (10 mM) (orange). **C** Estimation of the percentage of α-helicity (according to Eq. 2) of αS (blue) and αS-CEL (orange) in the presence of SDS micelles and the different SUVs. **D** Overlapping of the far-UV CD spectra of αS (20 µM) in the absence (black) and in the presence (blue) of ESC-SUVs (5 mM) on that corresponding to αS-CEL (20 µM) in the presence of ESC-SUVs (5 mM) (orange). All the CD spectra shown in the panels B and D were recorded in 20 mM phosphate buffer (pH 7.4) containing 150 mM NaCl and at 25 ºC

Human α-synuclein (αS) is a small monomeric and intrinsically disordered protein (IDP) mainly found in the presynaptic terminals of dopaminergic neurons. Its sequence contains three different domains: (i) a cationic (rich in Lys) N-terminal domain (M1-K60); (ii) a central domain (E61-V95)—known as the non-amyloid-β (NAC) domain; and (iii) a C-terminal acidic domain (K96-A140) (Fig. 1A) [1]. Their different physicochemical features, together with their conformational plasticity, have made αS to be part of several molecular machineries acting in the intraneuronal space. αS regulates the neuronal redox balance [2], inhibits apoptosis [3], stabilizes the glucose levels, and modulates the calmodulin activity [4].

Among all these physiological functions highlights its ability to regulate the homeostasis of synaptic vesicles (SVs) during the neurotransmitter release. This explains its subcellular localizations at the synapse [5], within the reserve pool of vesicles [6], in the mitochondria [7] or in the endoplasmic reticulum [8]. In fact, two of the few alterations shown by αS knock-out mice are modifications in the SVs pool size, and the reduction of the striatal dopamine [9, 10]. Hence, αS modulates the vesicle pool size and its mobilization [11], but it also regulates the vesicular endo- and exocytosis [12], which is essential for a correct neuronal crosstalk.

The participation of αS in the vesicle trafficking is related to its ability to bind them [13–15]. This interaction induces the vesicle clustering, which is a critical step in many biological events, such as the endoplasmic reticulum-to-Golgi vesicle transferring, or the recycling of the vesicles during the neuronal communication [13]. Hence, the binding of αS to synaptic membranes and its regulating capacity of their curvature along the endo- and exocytosis, become crucial for the correct neurotransmission.

Given the biological relevance of this process, evolution has delicately tuned the binding equilibrium of αS to lipid membranes [16]. In fact, the strength of its interaction with: (i) the presynaptic plasma membranes (during the endo- and exocytosis) [17]; (ii) the SVs [18]; and (iii) the inner mitochondrial membrane [19], depends on their lipid compositions. αS binds to phosphatidylserine [18], the main negatively charged lipid of SVs (~ 12%) [20], but it does not interact with phosphatidylcholine or phosphatidylethanolamine, the two main lipids found in the SVs (~ 60%) [20]. Likewise, αS interacts with cardiolipin [19], an anionic phospholipid found in the mitochondrial membrane [21]. Consequently, the αS-vesicle/membrane binding is promoted by electrostatic interactions between the anionic polar heads of some lipids, and the seven positively charged imperfect repeats (KTKEGV) found at the N-terminal domain of αS [14–22]. Their anchoring enhances the cooperative binding of the NAC domain, which modulates the overall affinity of αS to cellular membranes. However, it has no effect on the C-terminal domain, which remains detached from the lipid surface [15] acting as scaffold to recruit proteins to the membrane [23]. The binding of αS is also promoted by (i) hydrophobic interactions of the regions embedded in the hydrophobic core of the bilayer [18]; (ii) a high percentage of unsaturated aliphatic chains [24]; (iii) the vesicle curvature [25]; and (iv) packing defects in the bilayer [26].

The conserved amphipathic patterns of the first two domains of αS are responsible for a binding-induced conformational transition from their native unfolded states to α-helical conformations. These α-helices display an apolar side embedded in the bilayer, and an exposed hydrophilic face. Nonetheless, their exact α-helical structure is dictated by the membrane topology. Both domains fold into two broken antiparallel helices upon binding to micelles [27–29], whereas they do it into two separate helices and into an extended single helix when they bind SUVs mimicking SVs (Fig. S1) [14, 30].

The binding of αS to lipid bilayers might also have pathological implications related to its aggregation propensity [31]. αS can form oligomers that further evolve into amyloid fibrils and Lewy bodies (LBs) [32]. LBs are associated with the death of dopaminergic neurons [33] and the development of Parkinson’s disease (PD). Monomeric and oligomeric αS, as well as their fibrillar assemblies, can spread in a prion-like manner between neighbouring neurons. Once they bind to the neuronal membrane, they are internalized, and taken to the endosome. Its low pH (5–6) [34] could stimulate the formation of other assemblies, which might also escape from the cytoplasm spreading the αS pathology [31, 35]. The formation of the αS-SVs complexes could also accelerates the αS aggregation. However, this seems to be dependent on the composition of the SVs. While 1,2-dilauroyl-sn-glycero-3-phospho-l-serine (12:0) stimulates the aggregation of αS, a longer acyl chain (1,2-dimyristoyl-sn-glycero-3-phospho-l-serine; 14:0) reduced its aggregation rate, which become negligible in presence of 1,2-dioleoyl-sn-glycero-3-phospho-l-serine (18:1) [36, 37].

In addition to lipid interactions, there are other factors that rouse the aggregation of αS, such as (i) an increased expression of the αS encoding gene [38]; (ii) mutations (e.g. A53T, A30P, E46K or G51D) [39]; (iii) the formation of αS-metal complexes [40]; or (iv) post-translational modifications (PTMs) like nitration, truncation or oxidation [31, 41]. Moreover, αS can also be found non-enzymatically glycosylated in vivo. This process, known as glycation, arises from the reaction of its Lys with the oxidative by-products of the glycolysis, and it ends with the formation of a heterogeneous set of compounds known as advanced glycation end-products (AGEs) (Fig. S2). AGEs change the chemical nature of Lys and modify the physicochemical features of proteins. The accumulation of AGEs on LBs has been found to be really important to people suffering from diabetes mellitus [42, 43], which could explain why diabetes stimulates the development of PD [44–46]. The two most prevalent AGEs found on soluble monomeric and oligomeric αS, as well as on insoluble LBs, are the methylglyoxal-lysine dimer (MOLD) and N^ε^-(carboxyethyl)lysine (CEL) (Fig. S2) [47, 48]. Both arise from the reaction of αS with methylglyoxal (MG), a side product of the intraneuronal glycolysis [49]. MG facilitates the accumulation of αS oligomers [47] and diminish the protective role of αS against oxidative stress [50].

Despite the relevance of glycation in the context of PD there are very few studies reporting its precise effect on the conformation, the function, and the aggregation of αS. This has been hampered by the formation of a heterogeneous set of AGEs and a heterogeneous mixture of αS molecules with different glycation degree [50, 51]. To overcome this issue, we synthetized a modified αS where all its Lys were replaced by CEL (αS-CEL). Its study allowed us to prove that CEL extends the averaged unfolded conformation of αS as a result of the loss of its transient N-/C-terminal electrostatic interactions. In addition, we also proved that CEL inhibits the aggregation of αS, which indicates that the formation of CEL on LBs must be a later event after aggregation [51].

However, the most relevant pathological implications of CEL formation on αS might not be described yet. Given the relevance of the cationic Lys for the αS-SVs complex assembly, we hypothesized that their glycation-induced replacement by CEL could affect to their binding, to the vesicle clustering, and to the neurotransmitter release [52]. Here, we have applied different biophysical techniques to deeply study whether CEL interferes onto the binding of αS to SUVs mimicking SVs, and onto the αS-induced vesicle fusion. Our study provides clear experimental evidences on how a specific AGE (i.e. CEL) is able to modify the most biologically relevant function attributed to αS.

## Materials and methods

### Chemicals and reagents

1,2-Dioleoyl-sn-glycero-3-phosphoethanolamine (DOPE), 1,2-dioleoyl-sn-glycero-3-phospho-l-serine (DOPS) and 1,2-dioleoyl-sn-glycero-3-phosphocholine (DOPC) (Fig. S3) were purchased from Avanti Polar Lipids. All the other chemicals and reagents were analytical grade and they were purchased either from Sigma-Aldrich or from Acros Organics. All of them were used as received without further purification. All solutions used in this study were prepared using milli-Q water.

### Human α-synuclein expression and purification

Recombinant human α-synuclein (αS) was produced as we described before [50, 51]. In brief, *E*. *coli* BL21(DE3) transformed cells were grown in sterilized LB (25 g/L) containing ampicillin (100 μg/mL) at 37 ºC and 180 rpm. Cells were also grown in sterilized M9 media supplied with ^15^NH_4_Cl and ^13^C_6_-glucose as the only sources of nitrogen and carbon, respectively. This allowed to obtain ^15^N- and ^13^C-labelled αS. At OD_600nm_ = 0.6–0.8, αS expression was induced with IPTG (1 mM) and further incubated during 4 h at 37 ºC and 180 rpm. Afterwards, cells were centrifuged and the resulting pellet was resuspended in lysis buffer (10 mM Tris–HCl, 1 mM EDTA, 1 mM PMSF, pH 8.0) and stirred for 1 h at 4 ºC. Cells were then lysed and the cellular debris were removed by centrifugation. Nucleic acids were removed by adding streptomycin sulphate (1% *w*/*v*) and stirring for 1 h at 4 ºC, followed by centrifugation. The supernatant was supplied by the addition of (NH_4_)_2_SO_4_ (up to 0.295 g/mL) and additionally stirred for 1 h at 4 ºC. The obtained pellet was collected by centrifugation, dissolved in 10 mM Tris–HCl (pH 7.4) and filtered through a 0.22 μm filter. The obtained solution was loaded onto an anion exchange column (GE Healthcare RESOURCE™ Q; 6 mL) and αS was eluted with a NaCl gradient (0–600 mM). The purified protein was dialyzed into the desired buffer and stored at − 25 ºC until used. The purity of the obtained αS was checked using MALDI-TOF/TOF and SDS-PAGE electrophoresis. αS concentration was measured by UV–Vis spectroscopy using a molar extinction coefficient estimated on the basis of its amino acid content: *ε*_αS_280nm_ = 5960 M^−1^·cm^−1^.

### Chemical synthesis of N^ε^-(carboxyethyl)lysine (CEL) on αS

All the fifteen Lys of αS were chemically modified through the formation of CEL on them. The synthetic methodology was already described in previous works of our group, and it allowed to obtain an αS homogeneously modified with CEL (αS-CEL), which was characterized using different biophysical techniques [50, 51]. In brief, unlabeled or ^15^N,^13^C-labelled αS (100–200 μM) was incubated in the presence of pyruvic acid (50 mM) in 150 mM sodium phosphate buffer (pH 7.4) at 50ºC for 48 h. The reaction was carried out in the presence of 75 mM NaBH_3_CN, a reagent that selectively reduces the imine groups at neutral pH [53]. After 48 h of incubation, the reaction mixtures were dialyzed against phosphate buffer to remove the excess of the non-protein reagents. The synthesis of homogenous αS-CEL was confirmed by MALDI-TOF/TOF analysis, as the obtained mass spectrum displayed a narrow and unique peak (Fig. S4), which *m*/*z* was 1162 Da higher that the molecular weight of native αS. This proves that the fifteen Lys of αS and its N-terminal amino group were replaced by CEL moieties in αS-CEL.

### Lipid vesicle preparation

Lipid vesicles were freshly prepared just before performing each set of experiments. Initially, we took different aliquots from commercial stock solutions containing DOPE, DOPS or DOPC (25 mg/ml in CHCl_3_). These aliquots were then diluted in CHCl_3_ up to the desired concentrations. In addition, we also prepared a mixture containing the three different lipids DOPE:DOPS:DOPC at 5:3:2 molar ratio (ESC). Afterwards, the CHCl_3_ was removed using a rotary evaporator that worked under reduced pressure (290 mbar) and at 30 °C. The obtained lipid films were kept under vacuum during an extra hour. Finally, the films were hydrated for 1 h with a previously degassed 20 mM phosphate buffer (pH 7.4), which also contained 150 mM NaCl (from now named as buffer B1). The resulting solutions were used to prepare multilamellar vesicles (MLVs) by vortexing the lipid suspensions during 10 min. The obtained MLVs were then used to prepare small unilamellar vesicles (SUVs). MLVs solutions were first freeze at − 20 °C and thaw in a water bath at 37 °C with thorough vortexing for five times. Then, they were extruded 15 times through a 50 nm-pore size polycarbonate filter using the Avanti Polar Lipids mini-extruder.

The quality of the final SUVs (diameter and polydispersity index) was determined by Dynamic Light scattering (DLS) using a Zetasizer Nano instrument (Malvern Instruments, Malvern, UK). The obtained SUVs had a relatively uniform size with an average diameter of ~ 45 nm (Fig. S5). Additionally, the SUVs were further characterized using small-angle X-ray scattering (SAXS). These experiments were performed on a Xeuss 2.0 instrument (Xenocs, France) equipped with a microfocus Cu *K*_*α*_ source (*λ* 1.54 Å) and a Pilatus 300 k detector (Dectris, Switzerland). The distance between the detector and the sample was calibrated using silver behenate and it was set at 600 mm. The measurements were carried out for 2 h under vacuum at 25ºC using a low-noise flow cell. All data processing and fitting was carried out using the SasView software. The data were fitted using a recently developed slab model (*lamellar_slab_APL_nW*) [54], which describes lyotropic lamellar phases such as lipid bilayers. Fits were performed as single fits in the *q* range of 0.01–0.40 Å^−1^ using the *DREAM* algorithm as the fitting engine, which allowed to obtain the thickness of the overall bilayer, as well as that of its head group and its hydrophobic core (Fig. S6 and Table S1). The lipid concentrations in the solutions containing SUVs were determined using the Stewart’s method [55]. SUV solutions were stored at 4 °C until used.

### Circular dichroism spectroscopy

All CD data were recorded on a Jasco J-815 CD spectropolarimeter (Jasco, Gross-Umstadt, Germany) equipped with a temperature-controlled cell holder. The CD spectra of solutions containing 20 µM αS or αS-CEL were acquired at 25ºC in the absence or in the presence of (i) 10 mM sodium dodecyl sulphate (SDS) micelles; (ii) 5 mM DOPC-SUVs; (iii) 5 mM DOPS-SUVs; and (iv) 5 mM of ESC-SUVs. In addition, the CD spectra of samples containing 20 µM αS or αS-CEL in the presence of 10 mM SDS were acquired at 10, 20, 30, 40 and 50 ºC. All these mixtures were prepared in buffer B1. Control spectra were obtained from solutions containing SDS micelles or SUVs in the same buffer. All the spectra were acquired using a 1 mm-path length quartz cuvette, from 199 to 260 nm at 0.5 nm intervals and using a bandwidth of 1 nm. The scanning speed was 50 nm/min (2 s response time). The spectra were averaged from 10 accumulations.

The experimental scans were buffer subtracted, baseline corrected, and smoothed using a Savitzky–Golay smoothing filter. The ellipticity measured by the spectropolarimeter (*θ*, mdeg) was converted to mean residue ellipticity ([θ]_λ_, deg·cm^2^·dmol^−1^) according to Eq. 1.

1 $$\left[ \theta \right]_{\lambda } = \theta \times \left( {\frac{{0.1 \times {\text{MRW}}}}{l \times C \times 3298}} \right),$$

where *l* is the path length (cm), *C* is the protein concentration (mg/mL) and MRW is the protein mean weight per residue (g/mol), obtained from MRW = *M*/(*n* − 1), where M is the protein mean weight (g/mol) and *n* is the number of amino acids (140 for αS).

The α-helical content of αS and αS-CEL was derived in each case from the [*θ*]_222_ values according to Eq. 2.

2 $${\text{\% Helicity}} = 100 \times \frac{{\left[ \theta \right]_{222} - \left[ \theta \right]_{{{\text{coil}}}} }}{{\left[ \theta \right]_{{{\text{helix}}}} - \left[ \theta \right]_{{{\text{coil}}}} }}{ }.$$

The [*θ*]_222 nm_ values for the completely unfolded and completely folded proteins were obtained from the following Eqs. 3 and 4:

3 $$\left[ \theta \right]_{{{\text{coil}}}} = { 64}0 \, {-}{ 45}T,$$

4 $$\left[ \theta \right]_{{{\text{helix}}}} = \, - { 4}0000 \times \left( {{1 }{-}{ 2}.{5}/n} \right) + {1}00T,$$

where the *n* and *T* correspond to the number of amino acids in the protein and the temperature in degrees Celsius, respectively [56].

### NMR spectroscopy measurements

^15^N,^13^C-double-labelled αS (100 µM) or αS-CEL (230 µM) were used for NMR studies. These solutions were prepared in 20 mM sodium phosphate at pH 6.5 in the presence of 10% (v/v) D_2_O (from now named as buffer B2) and 40 mM d_25_-SDS.

^15^N-αS and ^15^N-αS-CEL (135 µM) were titrated using ESC-SUVs. Different aliquots from a stock solution containing 25 mM ESC-SUVs were added to 0.5 mL solution containing either αS or αS-CEL. We acquired the corresponding ^15^N-HSQC spectrum at each titration point (i.e. containing 0, 0.12, 0.25, 0.37, 0.62, 0.87 and 1.3 mM ECS-SUVs). All the solutions were prepared in buffer B2.

NMR experiments were recorded at 12.5, 20, 30 and 37ºC on a Bruker Avance III operating at a ^1^H resonance frequency of 600.1 MHz, and equipped with a 5-m ^13^C, ^15^N, ^1^H triple resonance cryoprobe. In all experiments, water suppression was achieved by the watergate pulse sequence [57] and proton chemical shifts were referenced to the water signal. ^13^C and ^15^N chemical shifts were referenced indirectly using the ^1^H,X frequency ratios of the zeropoint [58]. The spectra were processed using the software packages NMRPipe/NMRDraw [59] and Topspin (Bruker), whereas the data was analysed using Xeasy/Cara and Sparky.

### NMR assignment of SDS-bound αS and αS-CEL

The sequence-specific backbone assignment of αS and αS-CEL in the presence of d_25_-SDS, as well as the assignment of their side chain protons and carbons, were achieved at 37 ºC using different 2D- and 3D-NMR experiments: ^1^H,^15^N-HSQC, HNCACB, CACB(CO)HN, HNCO, HN(CA)CO, HAHN, ^15^N-TOCSY-HSQC, HCCH-TOCSY and CC(CO)NH.

Although the backbone assignment of the SDS-bound αS at 25 ºC was already published (BMRB code 5744) [27], here we have re-assigned these atoms at physiological temperature (37 ºC) and we have carried out the assignment of the side chains. At this temperature, the SDS micelles tumble more rapidly and most of the ^H^N-N cross-peaks can be observed, which does not occur at lower temperature (Fig. S7) [60]. The assignments of SDS-bound αS and αS-CEL were deposited in the BMRB data base under the accession codes 50,895 and 50,896, respectively.

The backbone chemical shift assignments were used to estimate the secondary structure content at residue level. This was carried out using different algorithms: (i) the neighbour corrected structure propensity calculator (ncSPC) [61], which bases its calculation on the ncIDP random coil library and adds an additional weighting procedure that accounts for the backbone conformational sensitivity of each amino acid type; (ii) the CSI 3.0 web server, which uses backbone chemical shifts to identify up to eleven different types of secondary structures [62]; and (iii) the TALOS + program [63], which uses the chemical shifts and to make quantitative predictions of the secondary structural content.

### NMR solution structure of the SDS-bound αS and αS-CEL

The solution structures of the SDS-bound fractions of αS and αS-CEL were calculated using the PONDEROSA-C/S package [64]. PONDEROSA-C/S comprises three different software: (a) PONDEROSA-Client that enabled the upload of the input data (i.e. the sequence; the NMR chemical shift assignments; the total ^13^C- and ^15^N-NOEs (Table S2); the dihedral angles obtained from PREDITOR [65] (Table S2); and the PDB models obtained from CS-Rosetta (*see the supplementary information for further details on the CS-Rosetta models*); (b) PONDEROSA-Server, which determines distance and angle constraints through ADUANA algorithm [66], calculates the 3D structures, and estimates the quality of the structures; and (c) PONDEROSA-Analyzer that allows to visualize the computed structures along with violations of input constraints and to examine/refine the restraints. After the first run, restraints were refined and they passed to PONDEROSA-Client for another round of structure calculation. Iterations were done until violations disappear in the final structures. To conclude the structure calculation, a final step was run using the “final step with explicit H_2_O” option, which provided the best 10 structures for αS and αS-CEL. PROCHEK-NMR [67] was used to analyse the quality of the structures through the Protein Structure Validation Server (PSVS) (https://montelionelab.chem.rpi.edu/PSVS/). The program Molmol was used to analyse the results and Pymol was used for structural representations. The structural ensembles of the micelle-bound αS and αS-CEL, as well as the average structures are provided as supplementary files.

### NMR diffusion experiments

The relative diffusion coefficients (*D*) of SDS-micelle bound αS and αS-CEL were measured from the diffusion-ordered spectroscopy (DOSY) spectra acquired using the pulse field gradient spin echo (PGSE) using a standard ledbpgp2s experiment [68]. Experiments were carried out at 10, 25, 37 and 45ºC on samples containing 100 μM αS or 230 μM αS-CEL, prepared in buffer B2 in the presence of 40 mM d_25_-SDS and 80 μM DSS. The experiments were collected using a diffusion time of 0.4 s and a length of the gradient pulse of 6 ms. For each spectrum we determined the *D* values for the ^1^H-NMR peaks appearing at 1.039, 1.108, 1.955, 3.829 (all these signals belong to the protein) and 0 ppm (a signal that belongs to the DSS and used as internal standard). The *D* value was obtained by fitting the intensity decays versus the gradient strength as described elsewhere [69].

### NMR relaxation measurements

^15^N longitudinal (*R*_1_) and transverse (*R*_2_) relaxation data, as well as steady-state ^15^N-HET-NOE data, were acquired for αS and αS-CEL at 37 ºC in buffer B2 in the presence of 40 mM d_25_-SDS. *R*_1_ values were determined using a series of 11 experiments with relaxation delays ranging from 10 to 2000 ms. *R*_2_ data was recorded using 11 different relaxation delays ranging from 8 to 112 ms. ^15^N-HET-NOE measurements were performed by 3 s high power pulse train saturation within a 5 s recycle delay. In all cases, relaxation and steady-state data were acquired using standard pulse sequences [70]. Recycle delays were 3 s in both *R*_1_ and *R*_2_ experiments. Sixteen scans in *R*_1_ and *R*_2_, and 32 scans in ^15^N HET-NOE spectra per *t1* experiment were acquired. 2048 × 128 complex points were obtained during *R*_1_, *R*_2_ and ^15^N-HET-NOE experiments.

### Dynamic light scattering (DLS)

DLS measurements of vesicle size distributions were performed using a Zetasizer Nano instrument (Malvern Instruments, Malvern, UK), and the data were analysed using the Malvern Zetasizer Software. The experiments were run at 90° scattering angle using a laser working at 633 nm. A viscosity of 0.9178 cP and a refractive index of 1.332 were used as parameters of buffer B1, and the material properties of the analyte were set to those of the lipids (absorption coefficient of 0.001 and refractive index of 1.450). SUVs were used at a concentration of 0.5 mM and the experiments were performed at 25 °C. For each measurement, accumulation of the correlation curves was obtained from 20 replicas. All the measurements were done in duplicate.

DLS measurements were also used to study the ability of αS and αS-CEL to promote the interaction and fusion of SUVs. Stock solutions containing SUVs of DOPC, DOPS or ECS (130 µM), in the absence or in the presence of 13 µM αS or αS-CEL, were prepared in buffer B1 and incubated during 96 h at 25 °C. Measurements were done after 0, 24, 48 and 96 h of incubation. The parameters used for each measurement were the same described above. The correlation curves were obtained after 20 replicas.

### Fluorescence anisotropy

Fluorescence anisotropy of 1,6-diphenyl-1,3,5-hexatriene (DPH) and 1,6-diphenyl-1,3,5-hexatriene-4′-trimethylammonium tosylate (TMA-DPH) was measured to determine the effect of αS and αS-CEL on the lipid ordering of DOPC-, DOPS- and ESC-SUVs. DPH is hydrophobic and it was used to monitor changes in the middle of the bilayer, whereas TMA-DPH maintains its polar region anchored at the membrane–water interface, allowing the study of the polar head groups order. Measurements were carried out on a Cary Eclipse fluorimeter (Varian, Palo Alto, CA, USA) equipped with Varian Auto Polarisers, with slit widths of 5 nm for both excitation and emission, and a Peltier controlled multicell holder. All data were acquired using a 10 mm-path length quartz cuvette. Stock solutions of DPH (125 µM) and TMA-DPH (250 µM) were prepared in dimethyl sulfoxide. SUVs were labelled with DPH or TMA-DPH through their incubation at 25 ºC in buffer B1 during 1 h with constant stirring [19]. The final concentration of lipid in the cuvette was 130 µM, and the final DPH and TMA-DPH concentrations were 1 µM and 2 µM, respectively. Stock solutions of αS and αS-CEL at three different concentrations (25, 100 and 200 µM) were prepared in buffer B1, and then titrated into suspensions of fluorophore-labelled SUVs to reach lipid/protein ratios of 500:1 (0.26 µM protein), 100:1 (1.30 µM protein) and 10:1 (13 µM protein). Control data were obtained from the titration of SUVs with buffer B1. The emission fluorescence of both fluorophores in buffer B1 was negligible.

DPH and TMA-DPH fluorescence anisotropy was measured at 25 °C after 5 min of incubation with constant stirring in the absence and in the presence of αS or αS-CEL. The excitation wavelength was set at 358 nm, with the excitation polariser oriented in the vertical position, while the vertical and horizontal components of the polarised emission light were recorded through a monochromator set at 410 nm. Each point was obtained from the average of five measurements. Experiments were done in duplicate. The anisotropy (*r*) of each sample was calculated from Eq. 5.

5 $$r = \left( {I_{{{\text{VV}}}} - G \times I_{{{\text{VH}}}} } \right)/\left( {I_{{{\text{VV}}}} + 2G \times I_{{{\text{VH}}}} } \right).$$

*I*_VV_ and *I*_VH_ are the parallel and perpendicular fluorescence intensity, respectively, and *G* is the ratio of the sensitivities of the detection system for the parallel (*I*_VV_) and perpendicular (*I*_VH_) polarised light. The *G* factor was determined for each sample separately. As the anisotropy of DPH and TMA-DPH are directly proportional to the degree of packing of the lipid chains in membranes, they can be associated with an order parameter (*S*). Hence, from the anisotropy value, the lipid order parameter (*S*) was calculated using Eq. 6 [71]:

6 $$S = \frac{{\left[ {\left( {\left( {1 - \frac{2r}{{r_{0} }}} \right) + 5\left( {\frac{r}{{r_{0} }}} \right)^{2} } \right)^{\frac{1}{2}} - 1 + \frac{r}{{r_{0} }}} \right]}}{{\left( {\frac{2r}{{r_{0} }}} \right)}},$$

where *r*_0_ is the fluorescence anisotropy in the absence of any rotational motion. We use the theoretical value of *r*_0_ = 0.390 for both fluorophores [72].

Fluorescence anisotropy measurements were also used to study the binding of αS or αS-CEL to SDS micelles and to SUVs. Fluorescence anisotropy of Y39 was recorded from samples containing 13 µM αS or αS-CEL in buffer B1, upon increasing the concentrations of DOPC-, DOPS- or ESC-SUVs (from 0 to 130 µM). Data were recorded at 25°C with an excitation wavelength of 280 nm, a bandwidth of 5 nm, and an integration time of 2 s. Emission values were recorded at 306 nm, which is the maximum emission wavelength of Tyr. Each data point is the average of 5 measurements. All the experiments were done in duplicate.

### Calcein efflux assay

DOPC-, DOPS-, and ESC-SUVs were filled with calcein through the hydration of the dried lipid films with a buffer B1 containing 50 mM calcein. This calcein solution was prepared dissolving the die in few microliters of 1 M NaOH, which were then diluted in B1 [73]. After 1 h of hydration, SUVs were prepared as described above. Unencapsulated dye was separated from the vesicles by gel filtration through a PD-10 Desalting Column packed with Sephadex G-25 Medium (GE Healthcare).

Time-dependent changes in the fluorescence intensity of 130 µM calcein-loaded SUVs, in the absence or in the presence of 13 µM αS or αS-CEL, were measured during 1 h on a Cary Eclipse fluorescence spectrophotometer (Varian, Palo Alto, CA, USA) using 96-well plates (*λ*_exc_ = 495 nm; *λ*_em_ = 515 nm). The maximal calcein leakage was obtained adding 1% Triton X-100 to samples containing 130 µM calcein-loaded SUVs.

## Results

### CEL decreases the ability of αS to adopt a micelle-induced α-helical conformation

Since the acquisition of an α-helical fold seems to be intimately related to the binding of αS to lipid aggregates [27–29], we initially used anionic sodium dodecyl sulphate (SDS) (Fig. S3) micelles as a lipid-mimetic to study whether CEL was able to modify the α-helical folding of αS.

The CD spectrum profile of αS-CEL in the presence of micelles was the typical of an α-helical structure. However, its ellipticity was remarkably lower than that shown by αS (Fig. 1B). Hence, the formation of CEL reduces in ~ 40% the α-helical content adopted by αS in the presence of anionic micelles (Fig. 1C).

### CEL abolishes the SUV-induced α-helical folding of αS

We then studied whether this reduction also occurred when using SUVs mimicking SVs. We used three different SUVs of similar size: (i) an anionic one formed by DOPS; (ii) a neutral one (used as control) containing DOPC; and (iii) one formed by a mixture of DOPE, DOPS and DOPC (5:3:2; ESC) (Figs. S3, S5, S6 and Table S1). DOPC-SUVs did not induce the α-helical folding of αS nor of αS-CEL (Fig. S8A). On the contrary, the CD spectra profiles of αS indicated that DOPS- and ESC-SUVs are able to fold αS into α-helical structures. However, these two SUVs were not able to fold αS-CEL, as its CD spectra profiles were still those characteristic of a random coil conformation (Figs. 1D, S8B). The inhibitory effect of CEL on the SUVs-induced folding of αS was really noticeable, as its α-helical content decreased in ~ 95% and in ~ 90% when DOPS- and ESC-SUVs were present, respectively (Fig. 1C).

Consequently, these results clearly prove that CEL abolishes the ability of αS to adopt an α-helical fold in the presence of the anionic DOPS- and ESC-SUVs.

### CEL depletes the affinity of αS towards SDS micelles

Although CD data prove that CEL precludes αS from adopting its lipid-induced α-helical folding, this does not indicate that the binding does not occur. In fact, several αS stretches retain a certain level of disorder even under its membrane bound states [14, 15], which could be also the case for the entire αS-CEL. Hence, we studied whether CEL blocked the αS-lipid binding or it only hindered its folding.

**Fig. 2:**
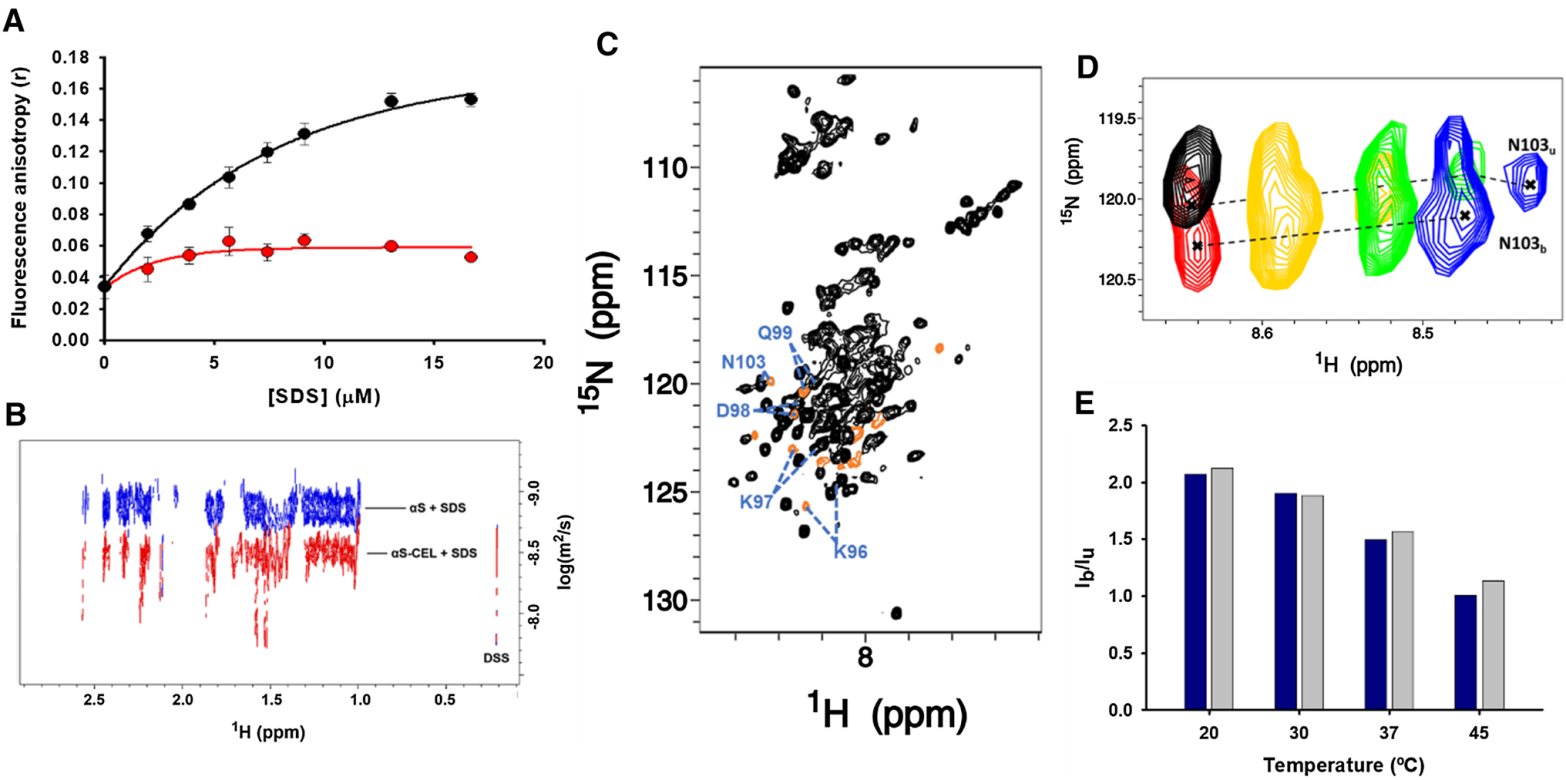
Studying the effect of CEL on the affinity of αS towards SDS micelles. **A** Fluorescence anisotropy (*r*) of solutions containing αS (13 µM; black) or αS-CEL (13 µM; red) at increasing SDS concentrations. All the measurements were carried out in 20 mM phosphate buffer (pH 7.4) containing 150 mM NaCl and at 25 °C. The continuous lines represent the theoretical function describing the fluorescence anisotropy change for αS and αS-CEL, which were obtained using Sigma Plot software. **B** Overlapping of the 2D-DOSY spectra of αS and αS-CEL. Both spectra were obtained at 45 °C in 20 mM phosphate buffer (pH 6.5) and in the presence of 40 mM d_25_-SDS. The spectra were referenced to the DSS signals. **C** ^1^H,^15^N-HSQC spectrum of αS-CEL in the presence of SDS micelles. Assigned signals are shown in black, whereas unassigned signals or those corresponding to unbound form of αS-CEL are coloured in orange. The C-terminal residues, of which we detected its bound and unbound resonances, are labelled in blue. The spectrum was acquired at 37 ºC in 20 mM phosphate buffer (pH 6.5). **D** Overlapping of ^15^ N-HSQC amide signals corresponding to N103 of αS-CEL in the absence (black signal) and in the presence of SDS micelles (red, yellow, green, and blue signals) at 12.5 ºC (black and red), 20 ºC (yellow), 30 ºC (green), and 37 ºC (blue). Dashed lines represent the temperature-dependent shifting of the resonances. The signals corresponding to the unbound residue are labelled as “N103u”, whereas those corresponding to the SDS-bound form are labelled as “N103b”. **E** Temperature dependence of the ratio between the intensities of the amide ^15^N-HSQC signals corresponding to the SDS-bound (Ib) and unbound (Iu) forms of N103 (dark blue) and D98 (grey) in αS-CEL

Initially, we collected the fluorescence anisotropy (*r*) of αS to evaluate the effect of CEL on its immobilization degree in the presence of SDS micelles. The *r* of αS is dominated by Y39, which is the only Tyr that interacts with micelles [28]. The *r* of αS rapidly increased as micelles were added up to an asymptotic limit, which indicates a complete change in the Y39 anisotropy. However, the *r* of αS-CEL scarcely changed during the addition of SDS (Fig. 2A), thus proving that the interaction of Y39 from αS-CEL with the micelles must be weaker than that displayed by Y39 of αS.

To determine whether this effect was a consequence of a CEL-induced change in the binding motif of αS or on the contrary, due to a CEL-induced decrease in its overall affinity, we acquired NMR-DOSY experiments, which enabled to compare the relative diffusion coefficients (*D*) of αS and αS-CEL in the presence of micelles. The *D* values of αS-CEL were higher than those of αS (Fig. S9), although their differences notably enhanced with temperature. This agrees with the markedly effect that the temperature had on the ellipticity changes of αS-CEL (Δ[*θ*]_222 nm_ = 0.04 deg.cm^2^/dmol·ºC and Δ[*θ*]_200 nm_ =  – 0.028 deg.cm^2^/dmol·ºC) in comparison to those shown by αS (Δ[*θ*]_222 nm_ = 0.03 deg.cm^2^/dmol·ºC and Δ[*θ*]_200 nm_ = − 0.018 deg.cm^2^/dmol·ºC), but also with the temperature-induced blue shift of their wavelengths at maximal ellipticity (Δ*λ* = 0.01 nm/ºC for αS and Δ*λ* = 0.045 nm/ºC for αS-CEL) (Fig. S10). Altogether, the CD and NMR-DOSY data prove that temperature has a higher effect on the unbinding equilibrium of αS-CEL than on that of αS, but also demonstrate that the micelle-bound fraction of αS-CEL, at physiological-like temperature (i.e. ~ 37 ºC), is lower than that of αS (Figs. 2B, S9, S10). Consequently, CEL makes the αS-micelle binding difficult. The presence of a remarkable percentage of unbound αS-CEL became evident from its HSQC spectrum. We detected several weak resonances arising from double conformational species, as they had the same spin system and unambiguous connectivities of other C-terminal residues (Fig. 2C). The chemical shifts of most of these weak resonances (at 12 ºC) perfectly matched with those of the corresponding residues of αS-CEL in the absence of SDS (also at 12 ºC) (Figs. 2D, S11) [51]. Thus, they can be unequivocally attributed to the presence of an unbound fraction of αS-CEL. Moreover, their intensities increased with the temperature while that of their micelle-bound counterparts diminished (Fig. 2E). Given that the exchange rate of the C-terminal amide groups of αS is electrostatically hindered [74] and it is not sensitive to temperature [75], we can ensure that the population of the unbound fraction of αS-CEL increases with temperature.

Our results prove that CEL reduces the affinity of αS towards the anionic SDS micelles, and demonstrate that αS-CEL, at physiological temperature, displays an equilibrium between the expected bound form and a remarkably populated unbound form.

### CEL hampers αS to bind SUVs

If CEL is able to reduce the affinity of αS towards the SDS micelles, it can be expected that it could have a similar effect on the interaction of αS with SUVs. In fact, the Δ*r* of Y39 in αS increased concomitant with the DOPS- and the ESC-SUVs addition up to the expected plateau, thus proving that Y39 binds to these SUVs [76]. However, the Δ*r* of αS-CEL was almost negligible during the addition of those SUVs, which indicates that Y39 of αS-CEL does not interact with them (Fig. S12).

**Fig. 3:**
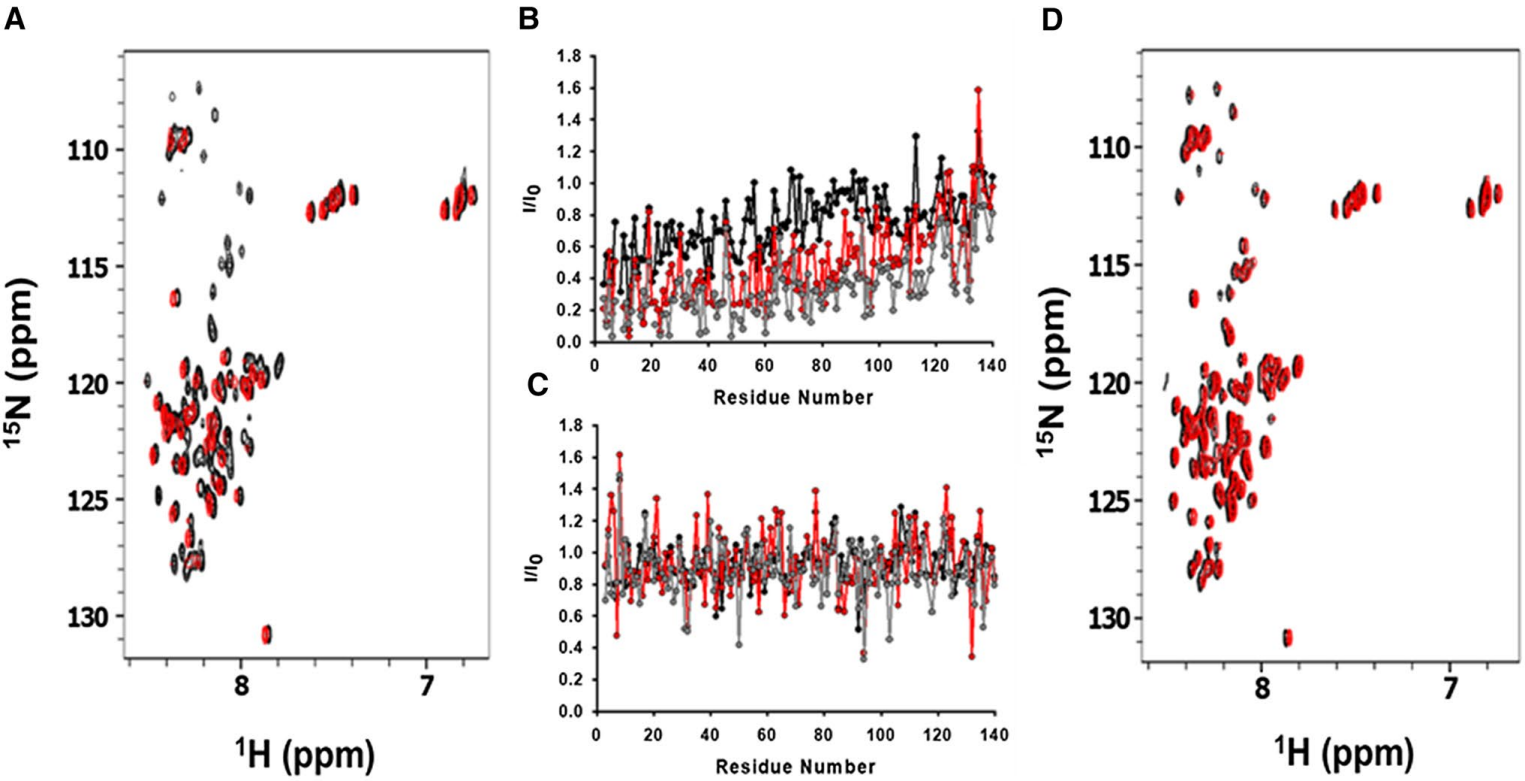
Impact of CEL on the affinity of αS towards ESC-SUVs. **A** Overlapping of the ^1^H,^15^N-HSQC spectra of αS (135 µM) before (black) and after (red) the addition of ESC-SUVs (1.3 mM). Both spectra were acquired at 37 ºC in 20 mM phosphate buffer (pH 6.5). **B**, **C** Fractional signal attenuation of the ^15^ N-HSQC signals relative to lipid-free spectra as a function of the residue number for αS (135 µM; **B**) and αS-CEL (135 µM; **C**) in the presence of ESC-SUVs at 250 µM (black), 610 µM (red), and 1.3 mM (grey) concentrations. **D** Overlapping of the ^1^H,^15^ N-HSQC spectra of αS-CEL (135 µM) before (black) and after (red) the addition of ESC-SUVs (1.3 mM). Both spectra were acquired at 37 ºC in 20 mM phosphate buffer (pH 6.5)

The effect of CEL on the affinity of αS to ESC-SUVs was additionally studied using NMR. A noticeable amount of the ^15^N-HSQC peaks of αS disappeared at a αS:ESC molar ratio of 1:10 (Figs. 3A, B, S13A) as a result of the signal broadening linked to the slow tumbling rate of the complex [76], thus proving the αS-SUVs binding. Nevertheless, the intensity of most of the ^15^N-HSQC peaks of αS-CEL did not change during the addition of ESC-SUVs (Figs. 3C, D, S13B), thus the main fraction of αS-CEL does not bind to SUVs (not at 37 ºC nor at 12 ºC). In any case, the chemical shifts of the ^15^ N-HSQC peaks still visible at protein:SUV molar ratios of 1:10 (Fig. S13) coincide with those displayed by αS and αS-CEL in the absence of SUVs [51]. This indicates that the unbound fraction of αS-CEL retains its random coil conformation, and that the region of αS that is not inserted into the SUVs (mainly the C-terminal domain) remains highly dynamic.

Hence, the replacement of Lys by CEL completely abolishes the ability of αS to bind anionic SUVs (DOPS), but also SUVs resembling SVs (e.g. ESC) [20]. Consequently, we have proved that CEL hampers the physiological functions of αS related to its ability to bind SVs or cellular membranes and therefore, the correct neuronal crosstalk.

### CEL scarcely change the structural features of the micelle-bound state of αS

Next, we aimed to understand what is the molecular mechanism that leads CEL to impede the αS-SUV binding. Is this because CEL hinders the folding need to embed αS into a hydrophobic core? Or is it only due to a CEL-induced loss of the electrostatic interactions tying αS to SUVs? These questions could be answered looking at the structure of αS-CEL once bound to SUVs. However, the low concentration of this complex and its NMR invisibility as a result of the signal broadening linked to its slow tumbling rate [76] precluded this study (Figs. 3, S13). Hence, we focused on the micelle-bound fraction of αS-CEL, which at 37 ºC seems to display a roughly equally populated equilibrium with its unbound counterpart. NMR allowed to selectively look at the micelle-bound states of αS and αS-CEL. This is because the intensities of the amide signals of the unbound αS/αS-CEL fractions decrease with temperature due to a fast exchange rate with the solvent (Fig. S7A), but also because the intensity of the signals of the αS/αS-CEL-bound states increases with temperature. This happens because micelles tumble more rapid, but also because the solvent exchange rate of the amide groups is slowed down due to the formation of α-helical-related hydrogen bonds and due to their insertion into the micelles (Fig. S7B). Accordingly, the intensity and the number of the ^15^ N-HSQC signals of αS and αS-CEL at 37ºC was much higher in the presence of SDS than in its absence (Fig. S14).

**Fig. 4:**
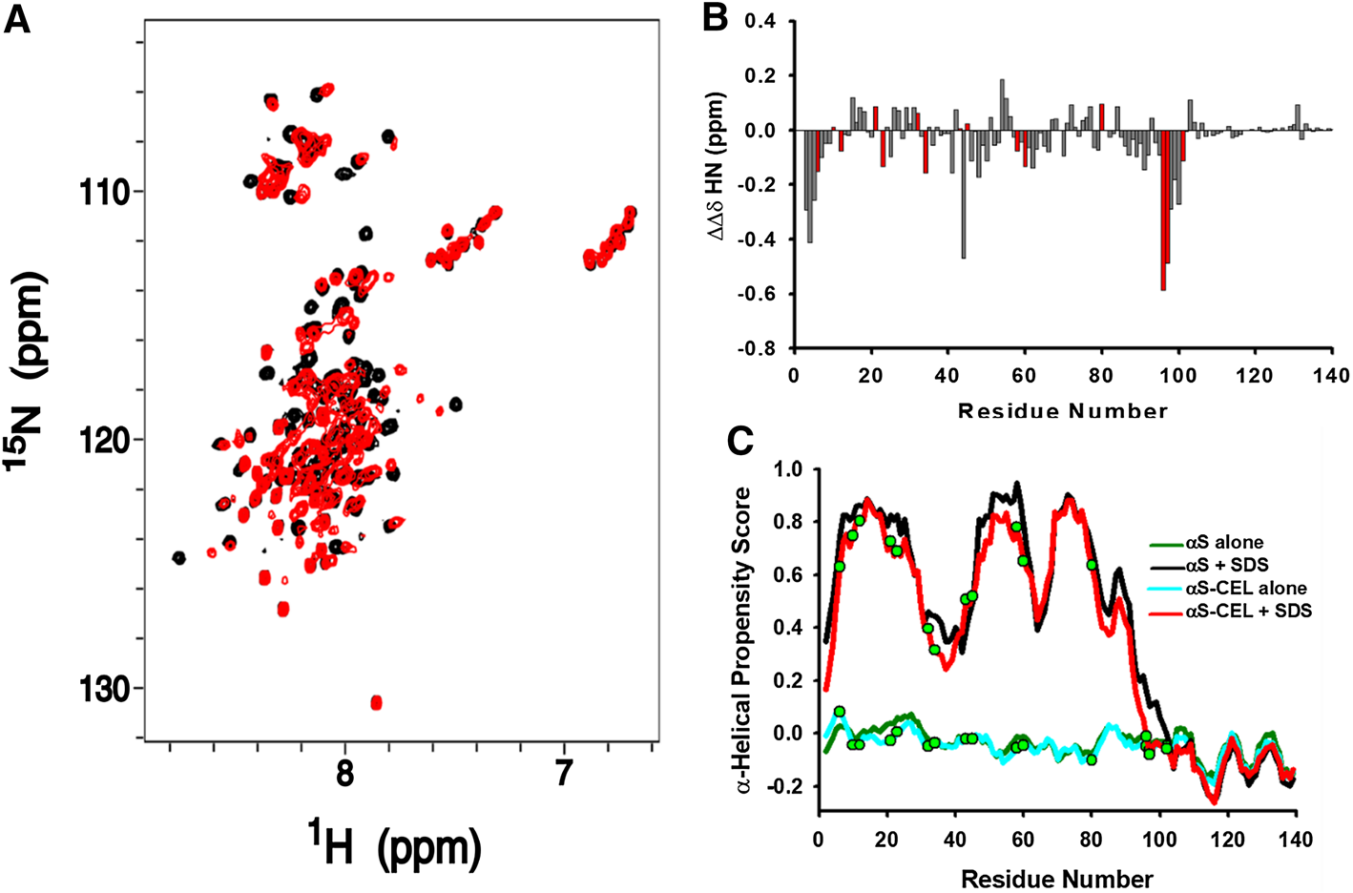
Using chemical shifts to unravel the effect of CEL on the structural descriptors of micelle-bound αS. **A** Overlapping of the ^1^H,^15^N-HSQC spectra of αS (100 µM; black) and αS-CEL (230 µM; red) in the presence of d_25_-SDS micelles (40 mM). Experiments were acquired in 20 mM phosphate buffer (pH 6.5) at 37 ºC. **B** Differences between the amide chemical shift perturbations (ΔΔ*δ*_HN_ = Δ*δ*_αS-CEL_—Δ*δ*_αS_) observed for αS and αS-CEL as a result of their interactions to SDS micelles (see Fig. S15). Bars corresponding to the experimental data obtained for the Lys/CEL residues are coloured in red. **C** Residue-specific ncSPC α-helical scores (https://st-protein02.chem.au.dk/ncSPC/) obtained for αS (black and green) and αS-CEL (cyan and red) in the absence (green and cyan) and in the presence (black and red) of SDS calculated from the HN, Hα, Cα, Cβ and CO chemical shifts. “+ 1” indicates the maximum propensity to form a full α-helix, “− 1” indicates a fully formed β-sheet, and “0” indicates disorder. The positions of the CEL moieties along the sequence of αS-CEL are shown as green dots

The ^15^N-HSQC spectrum of SDS-bound αS-CEL was different to that of αS (Fig. 4A), which indicates differences between their structures and/or their binding regions since we already proved that CEL formation per se does not have a direct effect on the backbone chemical shifts [51]. To understand those changes, we assigned the chemicals shifts of N, H_N_, C_α_, H_α_ and CO, as well as those of the H and C of the side chains of all residues between V3 and A140 in αS, but also in αS-CEL, with the exception of A11, T22 and T81. SDS addition induced a remarkable Δ*δ*_HN_ and Δ*δ*_CO_ perturbations on the same stretches of αS and αS-CEL (Fig. S15). Thus, the regions of αS and αS-CEL interacting with the micelles are roughly the same. Nevertheless, the binding-induced amide perturbation observed for the V95-K102 region in αS became negligible in the case of αS-CEL (Figs. 4B, S15).

The backbone chemical shifts were used to estimate the secondary structure content (Figs. 4C, S16). The micelle binding seems to induce the folding of αS-CEL into the two antiparallel helices typical of αS [27–29]. Hence, the structure of the micelle-bound αS-CEL must resemble that of αS. This folding was also confirmed by the presence of the characteristic HN,HN(*i*-1*,i*) NOEs observed for the N-terminal and NAC domains of both proteins (Figs. S17, S18A). In contrast, their C-terminal domains only shown the H_α_,HN(*i*-1,*i*) NOEs typical of extended conformations (Figs. S17, S18B), thus confirming that this domain does not undergo α-helical folding. αS and αS-CEL exhibited two stretches with a reduced α-helical propensity. One corresponds to the linker between the two helices (i.e. A30-T44) [27, 28], and the other is located at the NAC domain (i.e. T64-G88). This latter seems to be needed to endow the second helix with the curvature required to fit αS/αS-CEL onto the micelle surface [77].

**Fig. 5:**
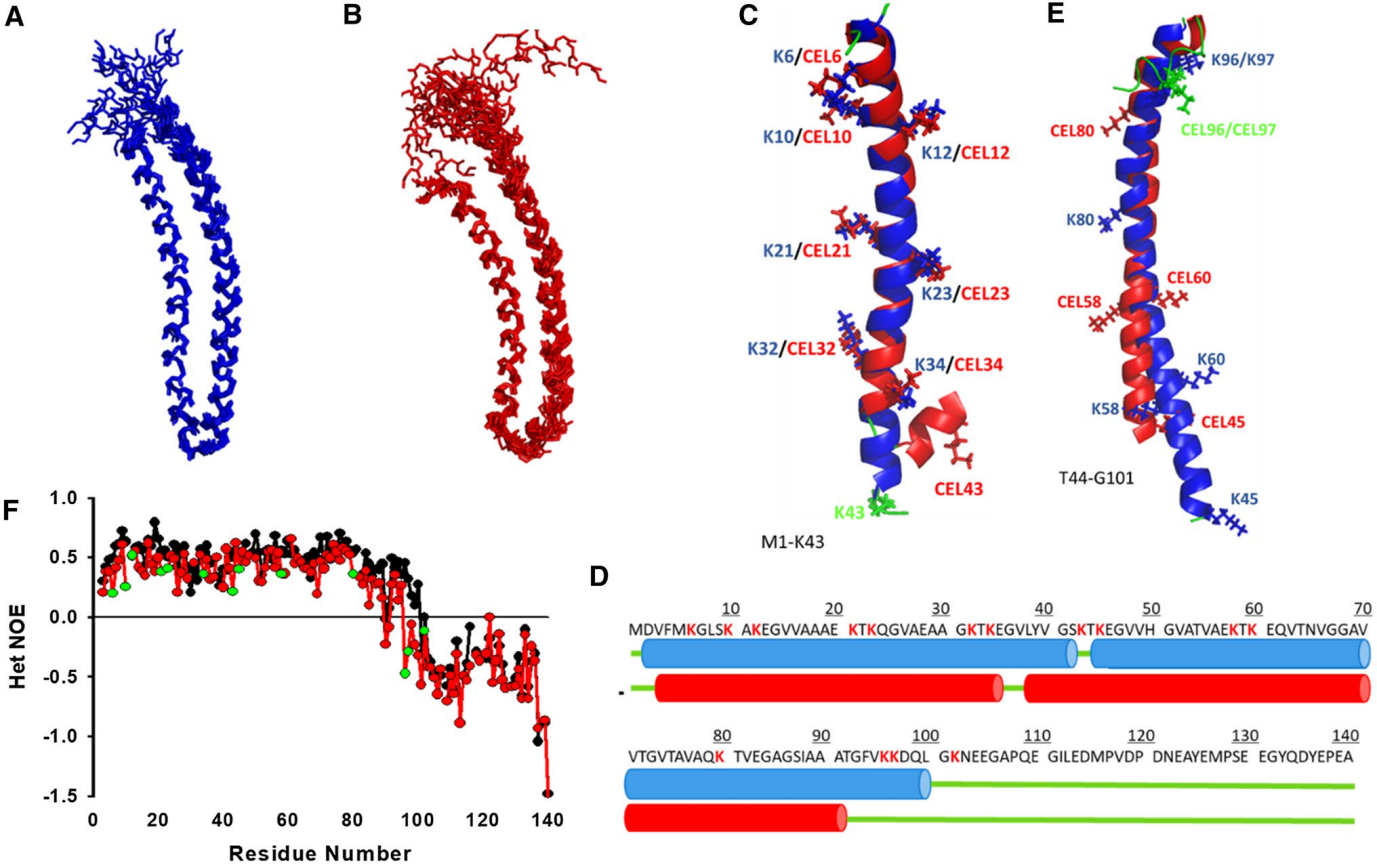
Structure and dynamics of SDS-bound αS and αS-CEL. **A**, **B** NMR bundles of the 10 lowest energy structures of αS (**A**) and αS-CEL (**B**). Traces connecting the backbone atoms are shown as sticks. In each case, the structures were aligned onto the V3-K43 stretch of the lowest energy structure (Table S2). **C** Cartoon representation of the structural alignment corresponding to the M1-K43 region (i.e. H1) of the averaged structures of αS (blue) and αS-CEL (red). The side chains of Lys (in αS) and CEL (in αS-CEL) are shown as sticks. Lys and CEL moieties are labelled according to its primary sequence numbering. The disordered regions are coloured in green. **D** Primary sequence of αS annotated for the secondary structure elements. α-Helices are indicated as blue cylinders and red cylinders in the case of αS and αS-CEL, respectively. Disordered regions are indicated as green lines. Lys in the primary sequence of αS are coloured in red. **E** Cartoon representation of the structural alignment corresponding to the T44-G101 region (i.e. H2) of the averaged structures of αS (blue) and αS-CEL (red). The side chains of Lys (in αS) and CEL (in αS-CEL) are shown as sticks. Lys and CEL moieties are labelled according to its primary sequence numbering. The disordered regions are coloured in green. The C-terminal region (G101-A140) lacks a well-defined secondary structure, and it is not shown to have a better view of the structured regions. **F** HET-NOE relaxation data obtained for αS (black) and αS-CEL (red) in the presence of SDS micelles. Experimental data corresponding to the different CEL residues are labelled in green. The relaxation data was acquired at 37 ºC in 20 mM phosphate buffer (pH 6.5)

We then used ^13^C-/^15^N-NOE-derived distance restrains, ϕ/ψ dihedral angles (Table S2) and CS-Rosetta models to calculate the structural ensembles of SDS-bound αS and αS-CEL. The representative families of the 10 lowest-energy structures of αS and αS-CEL were superimposable in the region of V3-K43 (Fig. 5A, B, S19) with low C_α_-RMSD values, and both had excellent Procheck scores (Table S2). The structure of the SDS-bound αS consists of an N-terminal α-helix (D2-S42; H1) connected to another α-helical stretch (K45-L100; H2), whereas the C-terminal domain (G101-A140) is disordered. The structure of the SDS-bound αS-CEL seems to be similar to that of αS (Fig. S20) although remarkable differences could be observed. CEL does not alter the helical structure of the first part of H1 in αS (D2-E35; C_α_-RMDS 1.087 Å), but it induces its breakage at G36-V37, thus shortening H1 (Fig. 5C, D). Consequently, H2 in αS-CEL starts at L38 instead of at K45 in αS, which is proven by the detection of several H_α_,HN(*i*,*i* + 3) and H_α_,HN(*i*,*i* + 4) NOEs between the residues of the L38-V48 stretch (Fig. S21). Hence, the positions of CEL43, CEL45, CEL58, CEL60 and CEL80 along the architecture of H2 are shifted respect to their Lys counterparts in αS (Fig. 5D, E). CEL formation on K96 and K97 could be the reason causing the shortening of H2 at the C terminus (it ends at T92 instead of at L100), thus turning the α-helical G93-L100 stretch into random coil.

Altogether, these data prove that the fraction of αS-CEL able to bind micelles adopts an α-helical structure resembling that acquired by αS, thus CEL does not impede the α-helical folding of the bound fraction. In addition, our structural data indirectly confirm that the CEL-induced loss of the electrostatic interactions between cationic Lys and anionic head groups of SUVs might constitute the main triggering factor shifting the binding equilibrium of αS-CEL towards the unbound form.

### CEL increases the local dynamics of the micelle bound structures

We also used the *R*_1,_ *R*_2,_ and HET-NOE data to analyse the dynamical properties of SDS-bound αS and αS-CEL. The relaxation values of the G101-A140 stretch in αS were remarkably lower than those of the D2-L100 region, thus confirming that the C-terminal domain is dynamic and it is not inserted into the micelles (Figs. 5F, S22). In addition, our relaxation data endorse our structural data since it confirms that the highly dynamic G101-A140 stretch in αS is expanded from G93 to A140 in αS-CEL. This proves that the region of αS-CEL embedded into the micelles is shorter (D2-G93) than that in αS (D2-L100). In addition, CEL slightly decreased the *R*_2_ and HET-NOE values of most of the Lys, and those of their neighbouring residues (Figs. 5F, S22B). This indicates that CEL increases the propensity of fast motions of these regions, which might be explained by the lower tendency of CEL to interact with micelles in comparison to cationic Lys.

These data demonstrate that, differently of what it is observed for αS, the G93-L100 stretch of αS-CEL remains highly dynamic, thus confirming that it is not embedded into the micelles. In addition, the higher mobility of the CEL moieties in comparison to Lys proves that CEL weakens the interaction pattern of the Lys with the polar head group of the micelles. Both findings contribute to mechanistically explain how CEL induces the shift of the equilibrium between the lipid-bound and unbound αS.

### CEL depletes the ability of αS to increase the lipid order degree of SUVs

**Fig. 6:**
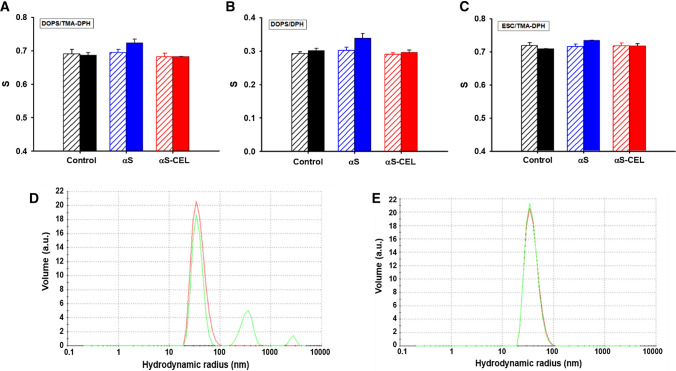
On the effect of CEL formation on the ability of αS to modulate the SUVs size and ordering. **A** Lipid order parameters (*S*) of DOPS-based SUVs (130 µM) labelled with TMA-DPH (2 µM) probe in the absence (black) and in the presence of αS (blue) or αS-CEL (red). **B** Lipid order parameters (S) of DOPS-based SUVs (130 µM) labelled with DPH (1 µM) probe in the absence (black) and in the presence of αS (blue) or αS-CEL (red). **C** Lipid order parameters (S) of ESC-based SUVs (130 µM) labelled with TMA-DPH (2 µM) probe in the absence (black) and in the presence of αS (blue) or αS-CEL (red). In the panels (**A**–**C**) empty bars represent the *S* values of the SUVs before addition of αS or αS-CEL. Full bars represent the *S* values of the SUVs after the addition of αS or αS-CEL (13 µM). **D**–**E** DLS size distributions of DOPS-SUVs (130 µM) before (red) and after (green) 96 h of incubation with αS (13 µM) (**D**) or αS-CEL (**E**). All the measurements were carried out in 20 mM phosphate buffer (pH 7.4) containing 150 mM NaCl and at 25 °C

Next, we investigated whether CEL could alter the ability of αS to modulate the lipid ordering in SVs and induce their clustering. We first used the fluorescence anisotropy of DPH- and TMA-DPH-labelled DOPS-, DOPC- and ESC-SUVs to compare the effect of αS and αS-CEL on the lipid order degree of these SUVs. The ordering of their acyls chains was the typical of a liquid-crystalline state, and it was higher in the polar region (*S* ~ 0.7) than in the hydrophobic core (*S* ~ 0.3) (Figs. 6A–C, S23). αS or αS-CEL did not change the ordering of the DOPC-SUVs (Figs. S23A, S23B), which can be ascribed to their low affinity. However, αS increased the order of the inner (Δ*S* ~ 12%) and the outer (Δ*S* ~ 5%) regions of DOPS-SUVs (Fig. 6A, B), whereas its effect on the ordering of the outer and the inner regions of ESC-SUVs was much lower and insignificant, respectively (Figs. 6C, S23C). This difference must be due to the lower affinity of αS towards ESC-SUVs, which have a negative surface charge density lower than that of DOPS-SUVs. In contrast, the presence of αS-CEL did not induce any change in the ordering of the lipid bilayers of these SUVs (Figs. 6A-C, S23C).

These results prove that CEL completely abolishes the capacity of αS to increase the lipid ordering in SUVs and to correct their defects. Consequently, CEL inhibits one of the most important physiological functions attributed to αS [18, 78].

### αS-CEL does not disrupt the packing of SUVs

It has been reported that αS oligomers are able to interact and permeabilize SVs [79, 80]. Consequently, we investigated whether monomeric αS-CEL had a similar membrane perturbation potential. The fluorescence intensity of calcein-loaded DOPS-, DOPC- and ESC-SUVs did not temporally change in the presence of monomeric αS nor of monomeric αS-CEL (Fig. S24). This proves that the lipid packing of these SUVs is not weakened by the presence of those proteins, thus CEL formation on αS does not impair the permeability of co-present SUVs.

### CEL formation abolishes the ability of αS to stimulate the fusion of SUVs

Another crucial physiological function attributed to αS is its ability to facilitate the clustering of SVs and assemble them into bigger vesicles [13]. Hence, we studied whether CEL formation affected this important function. Incubation of DOPC-, DOPS- or ESC-SUVs alone did not induce their self-fusion (Fig. S25). The presence of αS did not affect the size of DOPC-SUVs (Fig. S26A), but it promoted the interaction of DOPS- and ESC-SUVs, leading to notably bigger vesicle assemblies (Figs. 6D, S26B). In both cases we observed the formation of two new populations of SUVs, which size was ~ 10 and ~ 70 times bigger than that displayed by the same SUVs in the absence of αS (Table S3). Taking into account the overall percentage of the different SUVs obtained after 96 h of incubation, we can assert that the αS-induced fusion of DOPS-SUVs occurs faster than that of ESC-SUVs, which must be related to their different affinity towards αS. Contrarily, the DOPS- or the ESC-SUVs did not cluster when they were incubated with αS-CEL (Figs. 6E, S26C, S26D; Table S3), thus proving that CEL completely inhibits the αS-induced fusion of SUVs.

## Discussion

From the moment that mutations in the *SNAC* gene were found to be linked with the development of PD, αS became of high interest for neuroscience. Therefore, most of the studies focused on αS had the goal to understand its mechanistic role in the pathogenesis of PD. However, this knowledge is intimately related to the comprehension of its biological function. So far, the majority of its functions are linked to its ability to interact with cellular membranes. In fact, αS promotes the fusion of SVs with the plasma membrane [81], it is involved in the homeostasis of SVs during the neurotransmission [82], and it is able to regulate the pools of SVs [22]. Consequently, evolution has delicately fine-tuned the equilibrium between its membrane bound and unbound states [16], as its dysregulation would have fatal consequences in the neurotransmission.

The binding of αS occurs through ion-pair interactions between its highly conserved Lys (Fig. S27) and the anionic lipids, thus they are essential for a correct neurotransmission [83]. The number of Lys in the αS sequence is abnormally large (10.7%) and they are mostly located within the M1-K97 stretch (Fig. 1A). This region, although been natively disordered, lacks of the features of pure IDPs but also of the stability needed to fold. Hence, the metastability of its disordered and structured states gives this region a metamorphic plasticity that enables αS to undergo a binding-induced acquisition of different α-helical folds and consequently, to participate in multiple process [14]. Besides the role of the Lys in the interaction of αS with membranes, ten of them are targets for ubiquitination and SUMOylation [84], whereas others (K6 and K10) might be also acetylated [85].

Nonetheless, the Lys of αS hide a dark side. The nucleophilicity and exposure of their side chains turn them out as targets for endogenous aldehydes [52, 86]. Immunohistological studies proved that lipid peroxidation products (i.e. 4-hydroxynonenal, acrolein or malondialdehyde) [87, 88] are covalently bound to Lys of αS, and that 3,4-dihydroxyphenylacetaldehyde (a dopamine metabolite) can also modify them [89]. In addition, Lys-derived AGEs (MOLD and CEL; Fig. S2) have been detected on soluble monomeric [47] and oligomeric [48] αS, as well as on LBs isolated from PD patients [45], which must be related with the molecular mechanism behind the stimulating effect that diabetes has on PD [46].

So far, most of the scientific work studying these Lys-related PTMs has been focused in trying to understand how they modify the aggregation propensity of αS and the toxicity of the resulting aggregates [52, 86]. However, their effect on the physiological functions of αS and the implications that this would have on the pathogenesis of PD have been scarcely studied. Recently, we set the first step towards the precise comprehension of the effect of CEL on αS. We synthetized an αS were all its fifteen positively charged Lys were replaced by zwitterionic CEL moieties (αS-CEL) [51]. It is clear that our αS-CEL slightly overestimates the level of CEL modification in vivo, but not that much, since all the N-terminal Lys of the αS isolated from MG-treated rat, mouse and yeast were replaced by CEL [47]. The Lys of the NAC and C-terminal domains were less modified, but still between 66 and 87% of the Lys of those αSs sequences were replaced by CEL [47]. Nevertheless, our αS-CEL model overcomes the heterogeneity typically linked to protein glycation, but it also allows to study the effect of a specific AGE. CEL extended the average conformation of αS as a result of the loss of the electrostatic interactions tying the N-terminal and C-terminal domains, but it also inhibited the aggregation propensity of αS [51]. These results let us to predict that CEL formation would have a devastating effect on the biological functions of αS [51, 52]. To prove this hypothesis, we have now studied its influence on the ability of αS to interact with micelles and SUVs mimicking SVs.

CEL decreased the micelle-induced α-helicity of αS (in ~ 40%), and its effect was much more pronounced when using SUVs (Fig. 1B–D). The neutral DOPC did not induce any structuration on αS nor on αS-CEL (Figs. 1C, S8A), thus confirming that anionic charges at surface of SUVs are needed to fold αS. We also used the anionic DOPS- and ESC-SUVs. This latter nearly mimics the composition of SVs [20] and therefore, it has been largely used to simulate the interplay between αS and SVs [15, 76, 90]. Both SUVs induced a remarkable α-helicity on αS, but they were unable to fold αS-CEL (Figs. 1D, S8B). Although other Lys-related PTMs—such as K6-ubiquitination [91]—do not affect the folding of αS, the inhibitory effect of glycation on the lipid-induced α-helical folding was also observed from αS incubated with MG [47]. All this confirms the crucial role of the cationic Lys in the lipid-induced structuration of αS, which is also reinforced by the increase in the MLVs-induced α-helical content of αS concomitant with the number of the Glu-to-Lys replacements [92, 93].

It is likely that the loss of the lipid-induced structuration of αS occurs as a consequence of a CEL-induced shift of its binding equilibrium towards the disordered unbound conformation. This is the case of phosphorylated αS at Y39, but also when Y39 and/or Y125 are nitrated [94]. Despite that, we cannot rule out the possibility that αS-CEL binds lipids with a similar affinity than αS, but retaining its native disordered structure [51], as several B- and T-cell receptors (i.e. *ζ*_cyt_, FcεRIγ_cyt_, and CD3ε_cyt_) remain unstructured upon binding to anionic SUVs [95]. Consequently, we applied a set of different biophysical techniques to selectively analyse either the effect of CEL on the affinity of αS to micelles/SUVs, or the effect of CEL on the structure of its bound fraction.

Given that Y39 is the only fluorescent moiety reported to interact with lipids [28], we used its fluorescent anisotropy (*r*) change to qualitatively study the interaction of αS/αS-CEL with micelles/SUVs. The *r* of αS increased as micelles or SUVs were added. However, the addition of these lipids scarcely changed the *r* of αS-CEL (Figs. 2A, S12). Consequently, this suggests that the lower α-helical content of αS-CEL relative to that of αS might be related to a CEL-induced decrease in the population of the lipid-bound αS. However, we have also to take into account that CEL could not only change the affinity of αS, but also (or only) its binding motif. Thus, if Y39 in αS-CEL is within a stretch that has been detached from the lipids, its Δ*r* would be also negligible, even thought CEL would not affect the protein affinity. Alterations in the binding motif of αS have been associated with (i) the presence of Ca^2+^ (enhances the binding ability of the C-terminal domain) [96]; (ii) changes in the membrane architecture (the N65-K97 stretch does not bind the inner presynaptic membrane but it binds SVs) [17]; or (iii) PTMs (e.g. the phosphorylation of Y39 disrupts the binding of the V40-K97 stretch) [97]. Hence, the effect of CEL on the binding equilibria of αS was additionally studied using other biophysical techniques different from fluorescence.

NMR spectroscopy was used to measure the diffusion coefficients (*D*) of αS and αS-CEL in the presence of SDS micelles. The differences between the *D* values of αS-CEL and αS became larger as the temperature increased (Fig. S9). Accordingly, the temperature-induced changes in the wavelength of maximal ellipticity, in the [*θ*]_222 nm_ and in the [*θ*]_200 nm_, were always higher for αS-CEL than for αS (Fig. S10). Hence, all this proves that the temperature shifts the binding equilibrium of αS-CEL from is α-helical bound form towards its disordered unbound form, and it does it more pronounced than on the binding equilibrium of αS. Consequently, at physiological temperature, the population of micelle-bound αS-CEL is lower than that of αS. The noteworthy population of unbound αS-CEL at 37 ºC was confirmed through the assignment of several ^15^ N-HSQC signals arising from additional conformational species, which unequivocally belonged to the unbound αS-CEL (Fig. 2C). Hence, the depletion of the Lys-cationic charges seems not to be sufficient to completely break the micelle binding, but it has a clear effect on the strength of the interaction. Our findings agree with the results obtained by Bartels et al., who proved that the cationic M1-E20 peptide had a really low affinity towards anionic SUVs, thus indicating that positive charges are not enough for the membrane recognition [98].

Given that αS-CEL displayed a significant population of micelle-bound form, we investigated its structural features and compared them with those of the micelle-bound αS. NMR data collected at 37ºC allowed us to selectively look at these bound states (Figs. 4A, S14). The NMR assignments were used to derive several structural restrains, which let us to obtain the solution structures. The H1 and H2 helical regions of αS roughly match with those reported in other micelle-bound structures (i.e. PDB: 1XQ8 and 2KKW) [28, 29]. However, several unambiguous long-range NOEs force H1 to be in contact with H2, which does not occur in the published structures (Fig. S28). Although the N-terminal domain contains a large number of Lys (Fig. 1A), their replacement by CEL does not affect the α-helical folding of its first part (i.e. D2-E35; C_α_-RMDS 1.087 Å; Fig. 5C). However, CEL breaks its α-helicity at G36-V37, which could be due to the formation of CEL on K34. This is supported by the CEL-induced decrease in the α-helical content of this stretch (Figs. 4C, S16). Consequently, the second antiparallel helix in αS-CEL starts at L38 instead of at K45 in αS, but it does not become longer since it ends at T92 instead of at L100 in αS (Fig. 5D, E). The detachment of the G93-L100 stretch from the micelle in the bound form of αS-CEL was confirmed by the lack of SDS-induced chemical shift perturbations in this region (Figs. 4B, S15), by the decrease in its α-helical content (Figs. 4C, S16), and by the increase in its conformational dynamics (Figs. 5F, S22). Hence, CEL expands the dynamic (and unbound) C-terminal region of αS, thus the region of αS-CEL embedded into the micelles is shorter (D2-G93 in αS-CEL vs. in D2-L100 in αS). This change can be attributed to the replacement of K96, K97 and K102 by CEL moieties, which would hamper the interactions between this cationic region and the SDS. Our results prove that the fraction of αS-CEL able to bind micelles folds into an α-helical conformation resembling that adopted by αS, thus the depletion of the N-terminal cationic charges affects the strength of the interaction but not the binding-induced folding.

After the study of the effect of CEL on the αS/micelle equilibrium, we analysed its influence on the αS/SUVs interaction. Titration of ^15^N-αS with ESC-SUVs evidenced the disappearance of most of the ^15^N-HSQC signals (Figs. 3A, 3B, S13A), thus proving their binding since the slow-tumbling rate of the resulting complex precludes its NMR detection due to the line broadening of its signals [76]. Nevertheless, the intensity and the width of the ^15^ N-HSQC peaks of ^15^ N-αS-CEL did not change upon addition of ESC-SUVs (Fig. 3C, D, S13B) indicating that αS-CEL does not interact with ESC-SUVs. This behaviour was completely independent of the temperature (Figs. 3, S13), what shows that the disrupting effect of CEL on the αS-SUV binding is tougher than that exhibited onto the αS-micelle interaction. This might be due to the higher curvature and negative charge density of the SDS-micelles with respect to those in the ESC-SUVs, which stimulates the αS-lipid interaction [25, 77]. In any case, CEL formation on αS causes an effect comparable to the deletion of the M1-A11 segment, which drastically impaired the binding [99]. In addition, our NMR data prove that the inhibiting effect of CEL on the SUVs-induced α-helical folding of αS (Fig. 1C, D, S8B) is due to the lack of binding and not due to the binding of an unstructured αS-CEL.

Since CEL hampers the binding of αS to SUVs, it becomes evident that CEL will also have a disrupting role on the membrane organization events driven by the αS binding and consequently, on the neurotransmission. To prove this assumption, we have compared the effect of αS and αS-CEL on several structural features of DOPS- and ESC-SUVs. While αS slightly increases the ordering of their inner and outer regions, αS-CEL does not induce any modification on the lipid ordering (Figs. 6A–C, S23). Consequently, CEL takes away αS from one of its most important functions that is to correct the defects of SVs [18, 78]. Another important function attributed to αS is its ability to cluster [100] and fusion SVs within themselves [13] and with the neuronal membrane [12]. This seems to occur through a double-anchor mechanism in which a molecule of αS binds and bridges two different SVs [13]. αS stimulates the interaction of DOPS- and ESC-SUVs since in both cases, two new populations of bigger assemblies were formed (their size was ~ 10 and ~ 70 times bigger than that of the original SUVs) (Figs. 6D, S26B). Nonetheless, the formation of these big SUVs was not observed when the SUVs were incubated with αS-CEL (Figs. 6E, S26D; Table S3). Hence, CEL formation eliminates the ability of αS to cluster and fusion vesicles.

The toxic features that CEL formation on αS could have on the neurotransmission could go beyond its disrupting effect on the vesicle metabolism. Given that αS oligomers can alter the packing of SVs [79], we also studied whether monomeric αS-CEL could carry out the same effect. This process seems to play a major role in the pathogenesis of PD due to the impairment of membranous cellular structures [101]. However, we observed that monomeric αS-CEL was not able to unpack or increase the permeability of the DOPS- or ESC-SUVs (Fig. S24), thus the toxic role of this AGE on the αS-mediated neurotransmission could limited to its hampering effect on the αS-SVs binding and consequently, on the SVs-SVs and SV-membrane interactions.

The results we provide here, together with those previously published [51], allow to complete the puzzle explaining the mechanism by which the non-enzymatic modification of αS by CEL contributes to the neurodegeneration associated with PD. CEL extends the native conformation of αS as a result of the loss of the N-terminal cationic charges that transiently tie the N-/C-terminal domains [51]. The loss of these cationic Lys completely depletes the affinity of αS towards synaptic-like vesicles, making impossible their clustering and fusion and therefore, a correct neurotransmission. Beyond its effect on the vesicle metabolism, the reduction of the membrane-bound fraction must imply an increase of the concentration of αS in the cytoplasm. This effect would be stimulated by the inhibitory effect of glycation on the neuronal release of αS [47], but also on the its ubiquitination [47, 84] and on its clearance through the chaperon-mediated autophagy (CMA) pathway. CEL formed on the Lys of the CMA binding motif would hamper its recognition and clearance [102]. Nevertheless, this concentration increase might not result into an enhanced aggregation [103], since we proved that CEL inhibits the aggregation of αS, although it is unable to disassemble pre-existing αS aggregates. Thus, CEL found on LBs must be formed in a later event after aggregation [51].

Our work demonstrates that the comprehension of how glycation stimulates the development of neurodegenerative disorders cannot be achieved looking only at its effect on the aggregation, but it is also necessary to completely understand how it affects the physiological protein function.

## Conclusion

Here, we prove that CEL formation on αS diminishes its affinity towards SDS micelles at 37ºC. However, the fraction of αS-CEL that binds micelles still exhibits the two broken antiparallel helices typical of the micelle-bound αS, whit the exception of the G93-L100 stretch, which is detached from the micelle. The effect of CEL was more pronounced on the interplay between αS and SUVs, thus the strength of the disrupting effect of CEL on the lipid binding depends on the membrane geometry and composition. CEL completely inhibited the ability of αS to bind SUVs and therefore, it hampered the capacity of αS to correct the vesicle defects and to promote their clustering and assembly. Consequently, the results we provide here represent a mechanistic explanation on how a specific AGE modifies the most important biological function of αS. Hence, from now, the understanding of the glycation effect on neurodegeneration needs to be faced beyond its impact on the aggregation or on the toxicity of the resulting aggregates, but also on the function of the protein/s linked to the development of each neurodegenerative disorder.

### Supplementary Information

Below is the link to the electronic supplementary material.

Supplementary file1 (DOCX 3994 KB)

Supplementary file1 (PDB 1615 KB)

Supplementary file1 (PDB 1555 KB)

## Data Availability

The NMR assignments have been deposited in the Biological Magnetic Resonance Data Bank (BMRB) under the accession codes 50,895 (αS) and 50,896 (αS-CEL.) All the other data are available upon request.
