# Supplementary material for: Glycation of α-synuclein hampers its binding to synaptic-like vesicles and its driving effect on their fusion
Source: Cell Mol Life Sci. 2022 Jun 4;79(6):342. doi: 10.1007/s00018-022-04373-4 (PMC9167179; doi:10.1007/s00018-022-04373-4)
Supplement: Supplementary file 1 — Supplementary file1 (DOCX 3994 KB) [file 18_2022_4373_MOESM1_ESM.docx]

Supplementary Information for

**Glycation of α-synuclein hampers its binding to synaptic-like vesicles and its driving effect on their fusion**

Ana Belén Uceda, Juan Frau, Bartolomé Vilanova, and Miquel Adrover^*^

Institut Universitari d'Investigació en Ciències de la Salut (IUNICS). Institut d’Investigació Sanitària Illes Balears (IdISBa). Departament de Química, Universitat de les Illes Balears, Ctra. Valldemossa km 7.5, E-07122 Palma de Mallorca, Spain.

*Correspondence to: Miquel Adrover, University of Balearic Islands,

Phone: +34 971 173491; Fax +34 971 173426;

e-mail: miquel.adrover@uib.es

1.- Structure predictions using CS-Rosetta……………………………………2

2.- Supplementary figures…………………………………………………………3

3.-Supplementary tables…………………………………………………………33

4.-References associated to the supplementary information……....…….35

**Structure predictions using CS-Rosetta**

The CS-Rosetta server (https://csrosetta.bmrb.wisc.edu/submit) was used to obtain structural models of SDS-bound αS and αS-CEL. CS-Rosetta uses chemical-shift-constrained modelling to create a protein structure based on the prediction of backbone dihedral angles from the amino-acid sequence and the analogy of the experimental chemical shifts with those of characterized stretches/structures derived from PDB [1]. The backbone chemical shifts (C_α_, C_β,_ C, N, H_α_ and HN) were used as input to generate 3,000 structural models for αS and αS-CEL. The Cα-Root Mean Square Deviation (Cα-RMSD) was calculated for all the models with respect to the lowest energy structure. The CS-Rosetta run was deemed as successful when it achieved a Cα-RMSD around 2 Å for non-flexible regions for the ten lowest energy structures. The homologous structures were excluded from the PDB search.

The lowest energy models of αS and αS-CEL displayed the expected two α-helices (**Fig. S29**) [2,3]. The K45-T92 helix of αS had a remarkable structural similarity with that stretch in the published NMR structure (PDB 1XQ8; Cα-RMSD 3.85Å), although it was in closer contact with its N-terminal counterpart (**Fig. S29A**). This proximity was also observed for the lowest energy model of αS-CEL, which N-terminal helix (V3-G36) was structurally identical to the N-terminal α-helix of the published structure (PDB 1XQ8; Cα-RMSD 0.836Å) (**Fig. S29B**). However, the αS-CEL model shown a completely bended K45-T92 helix, which was broken at the two consecutive Gly (G67-G68). We then aimed to use these models as inputs to calculate the NMR solution structures of αS and αS-CEL. However, we did not use the lowest energy model of αS-CEL since it did not fit to the experimental data. The ^13^C-NOESY-HSQC spectrum of SDS-bound αS-CEL shown unambiguous NOEs between the H_γ_ of CEL12 and the H_β_ of V82 (**Fig. S30A**), which should not be observed according to the lowest energy model of αS-CEL (**Fig. S30B**). Instead, we used the model of αS-CEL with the second lowest energy, which fitted to this restrain (**Fig. S29C**).

**Supplementary Figures**


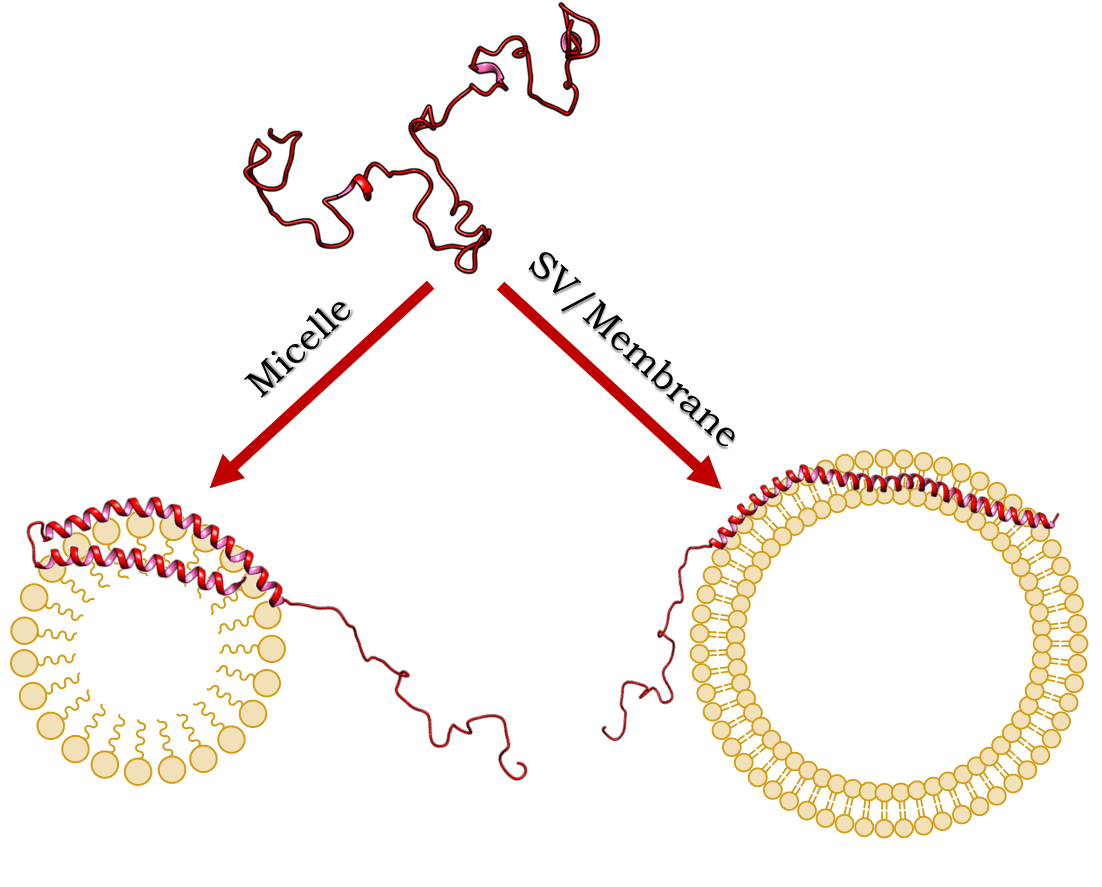


**Figure S1.** Model representing the folding process of αS (an intrinsically disordered protein under its unbound state) upon interaction with micelles (*left*) and with SUVs (*right*). The structure representing the averaged ensemble of native αS was obtained from the Protein Ensemble Database (PED9AAC) [4]. The structure of the micelle-bound state of αS was obtained from the Protein Data Bank (PDB code 1XQ8). The structure of αS under its SUVs-bound state was created using the micelle-bound state as a template (PDB code 1XQ8) followed by the manual modification of the dihedrals of the L38-T44 stretch (using the STRIDE software) [5] and the recalculation of the tridimensional structure using the PepMake (v 1.2.) software (http://pepmake.wishartlab.com/).

**Figure S2.** The overall mechanism of protein glycation and the formation of methylglyoxal-derived AGEs. Protein glycation starts with the chemical reaction of reducing carbohydrates (mainly glucose) with primary amino groups of proteins. This encompasses the reversible formation of a Schiff base that converts into an Amadori product, which can then further rearrange to yield the advanced glycation end products (AGEs). Although the formation of the Schiff base and the Amadori compound constitute the central pathways along the protein glycation mechanism, the whole process become much more complex as a result of collateral oxidative reactions of reducing sugars, Schiff bases and Amadori compounds [6]. These reactions yield highly reactive carbonyl species such as methylglyoxal (MG), which can further react with other amino acid side chains contributing to AGEs formation. MG is the most relevant glycating compound inside the neurons [7], and it is able to modify S through the formation of MOLD and Nε-(carboxyethyl)lysine (CEL) [8] on its Lys side chains.

**Figure S3.** Chemical structures of SDS, DOPC, DOPE and DOPS, which are the lipids used in this work to assemble the micelles (SDS) and the different SUVs that we have used to study the αS/αS-CEL-lipid interactions.


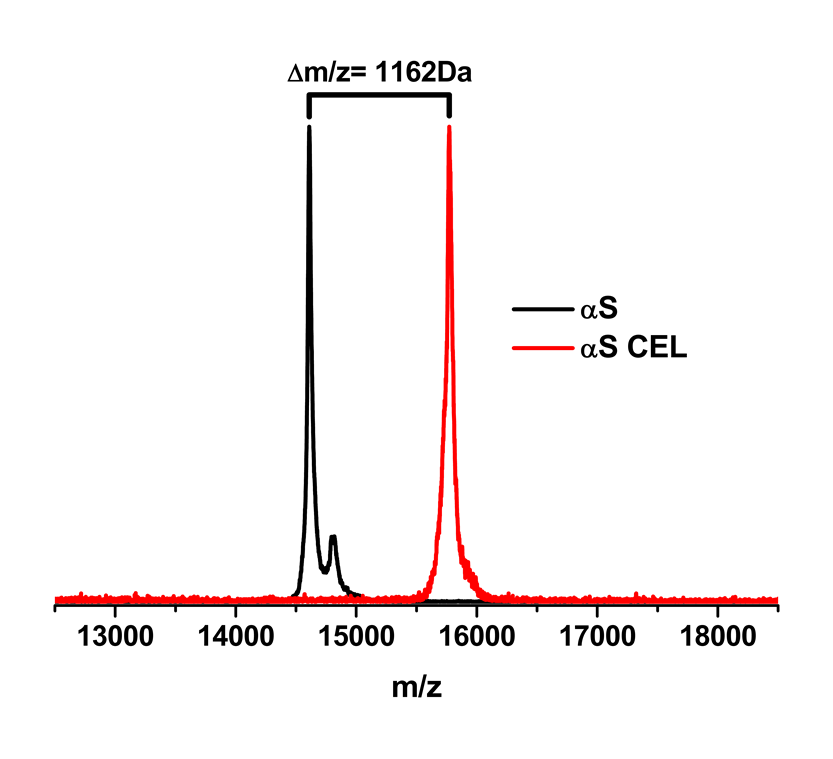


**Figure S4.** Overlapping of the MALDITOF/TOF spectrum of the native S with that corresponding to S-CEL. The intensity of the peaks was normalized for comparison purposes.


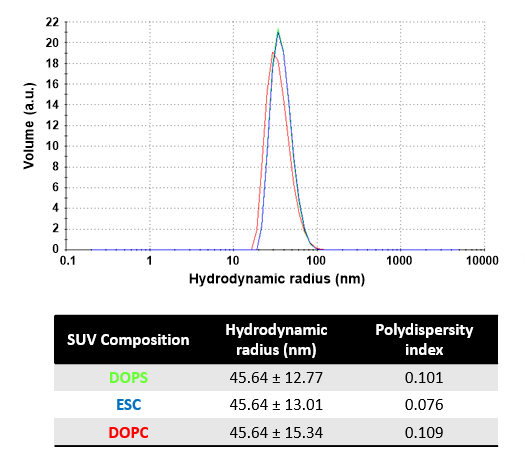


**Figure S5.** DLS size distributions profiles obtained for different solutions containing 130 µM DOPS-SUVs, (*green*), ESC-SUVs (*blue*) or DOPC-SUVs (*red*). All the experimental data was collected in 20 mM phosphate buffer containing 150 mM NaCl at pH 7.4 and at 25 ⁰C. The size distribution profiles were collected just after the extrusion. The averaged hydrodynamic radius and the polydispersity index of all these SUVs are indicated in the attached table.


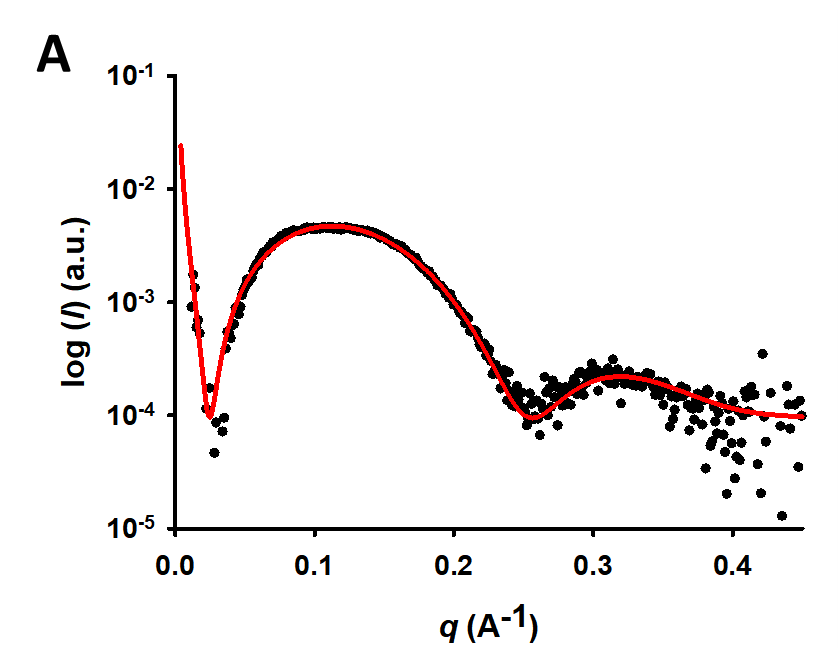


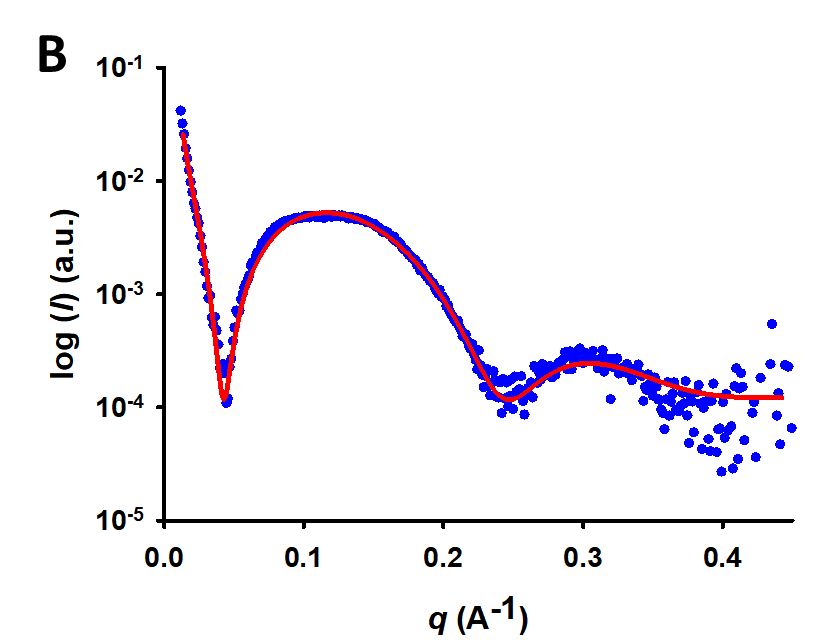


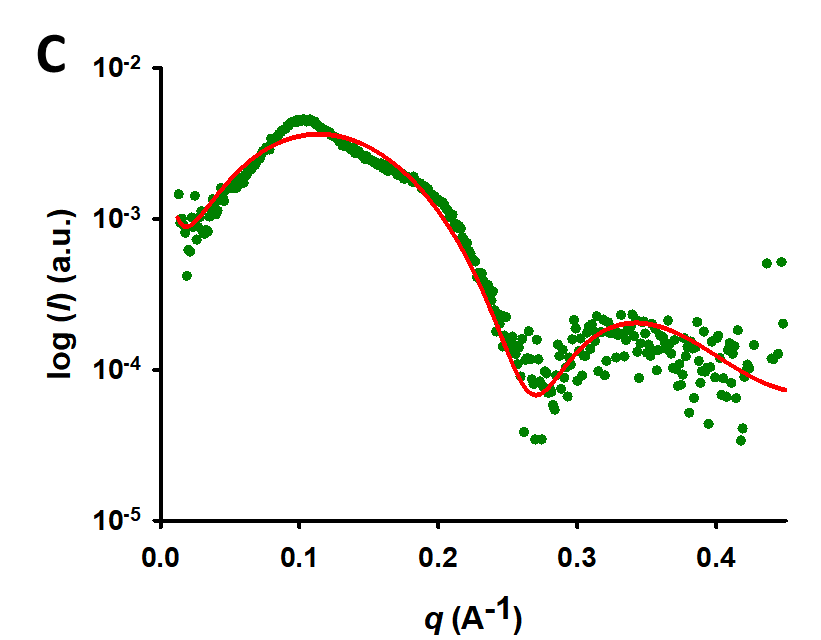


**Figure S6.** SAXS scattering curves obtained for **(A)** ESC-SUVs, **(B)** DOPC-SUVs and **(C)** DOPS-SUVs. The experimental data points are shown as dots, whereas their fitting to the *lamellar_slab_APL_nW* model are shown as red lines. The scattering curves have the typical shape already described for SUVs, with a pronounced maximum at high *q* and a shallow minimum at intermediate *q*, which typical of the asymmetry in the bilayer [9].


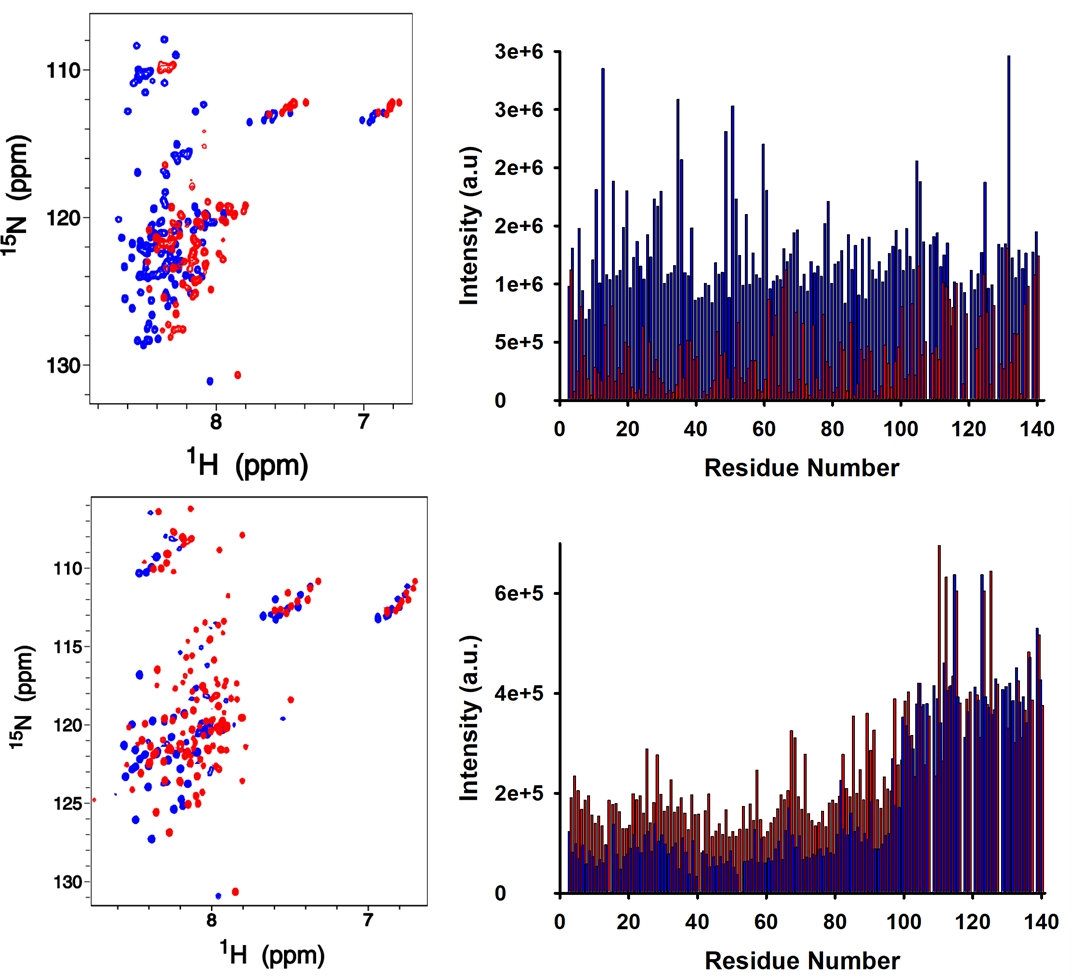


A

B


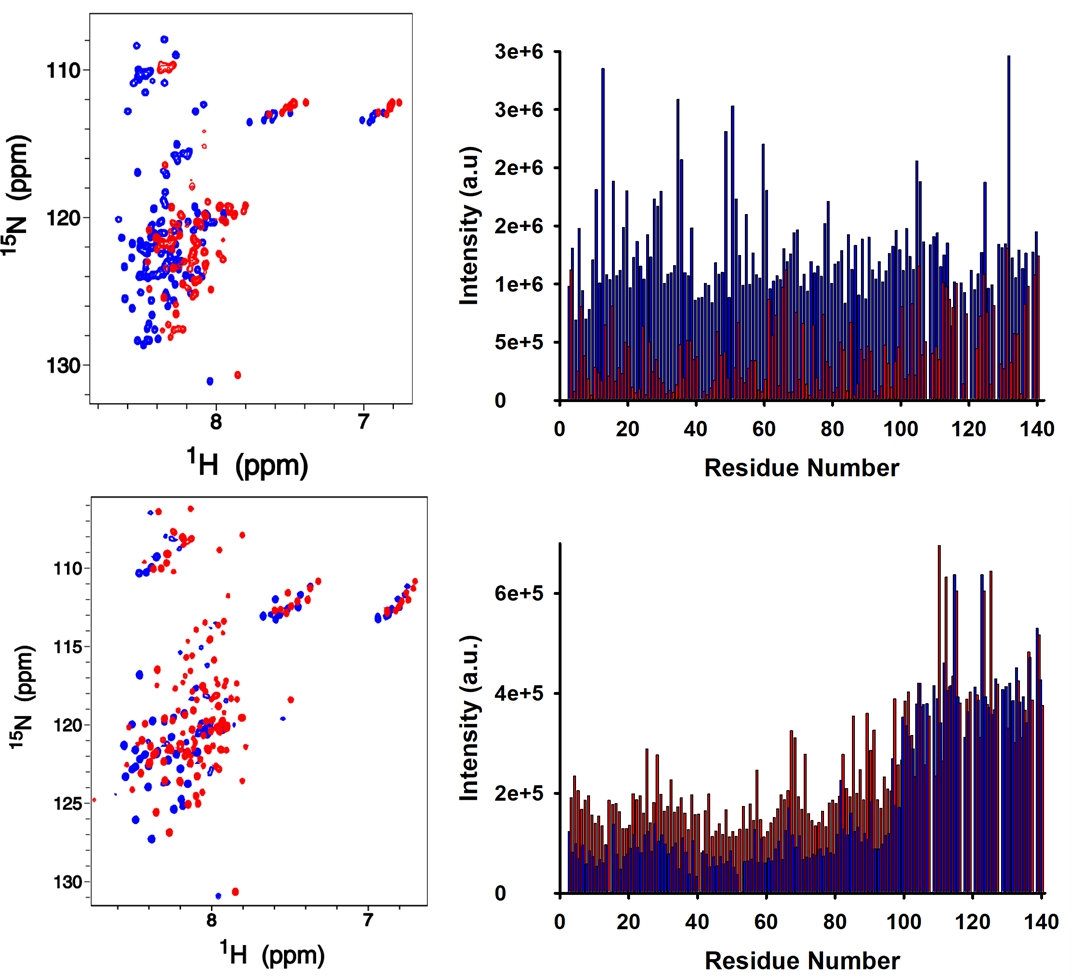


**Figure S7.** Effect of the temperature and of the presence of SDS on the intensities of the ^1^H,^15^N-HSQC signals of αS. **(A)** Left panel: overlapping of the ^15^N-HSQC spectra obtained for a solution containing αS (220 µM) at 12.5ºC (blue) and at 37ºC (red); right panel: intensity of peaks from the ^1^H,^15^N-HSQC spectra of αS at 12.5ºC (blue) and at 37ºC (red). **(B)** Left panel: overlapping of the ^15^N-HSQC spectra obtained for a solution containing αS (100 µM) and SDS (40 mM) at 25ºC (blue) and at 37ºC (red); right panel: intensity of peaks from the ^1^H,^15^N-HSQC spectra of αS in the presence of SDS at 25ºC (blue) and at 37ºC (red). All the spectra were acquired in 20 mM phosphate buffer at pH 6.5.


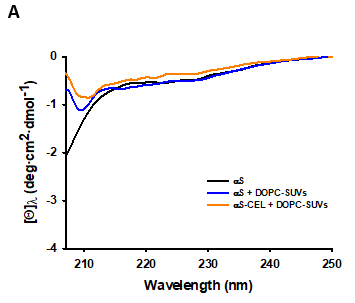


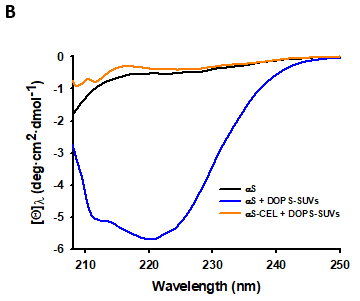


**Figure S8.** Circular dichroism study of the effect of CEL formation on the secondary structure adopted by αS in the presence of DOPS- and DOPC-SUVs. **(A)** Overlapping of the far-UV CD spectra of αS (20 µM) in the absence (*black*) and in the presence *(blue)* of DOPC-SUVs (5 mM) onto that obtained for αS-CEL (20 µM) in the presence of DOPC-SUVs (5 mM) *(orange).* **(B)** Overlapping of the far-UV CD spectra of αS (20 µM) in the absence (*black*) and in the presence *(blue)* of DOPS-SUVs (5 mM) onto that obtained for αS-CEL (20 µM) in the presence of DOPS-SUVs (5 mM) *(orange).* All the CD spectra shown in the figure were recorded in a 20 mM phosphate buffer (pH 7.4) containing 150 mM NaCl and at 25 ºC.

**Figure S9.** Differences between the averaged diffusion coefficients (*D*) of αS and αS-CEL (∆*D* = *D*_αS-CEL_-*D*_αS_) in the presence of SDS at different temperatures. For each temperature, four different ^1^H-signals appearing at 1.039, 1.108, 1.995 and 3.829 ppm were integrated, and their integrals were used to calculate the averaged differences between the *D* values at each temperature. All 2D-DOSY spectra were acquired in 20 mM phosphate buffer (pH 6.5) in the presence of 40 mM d_25_-SDS.


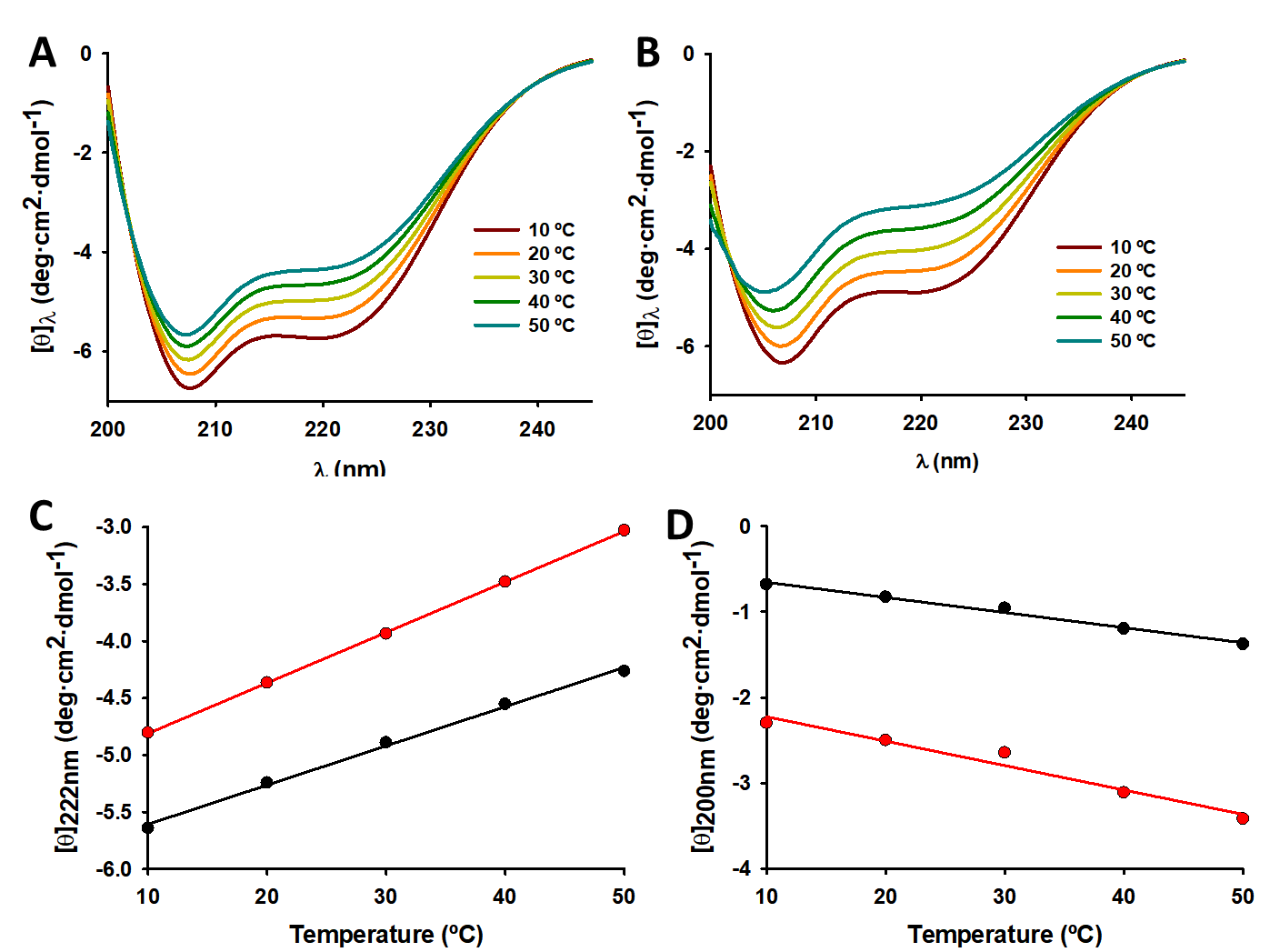


**Figure S10.** Effect of the temperature on the CD spectra of αS and αS-CEL obtained in the presence of SDS micelles. **(A-B)** Overlapping of the CD spectra obtained at different temperatures for solutions containing either αS (20 μM) **(A)** or αS-CEL (20 μM) **(B)** and SDS (10 mM). **(C-D)** Plots of the values of [Θ]_222nm_ **(C)** and [Θ]_200nm_ **(D)** collected at different temperatures for solutions containing either αS (*black;* 20 μM) or αS-CEL (*red;* 20 μM) and SDS (10 mM). Experimental data are shown as dots, whereas their fits to linear functions are shown as continuous lines.


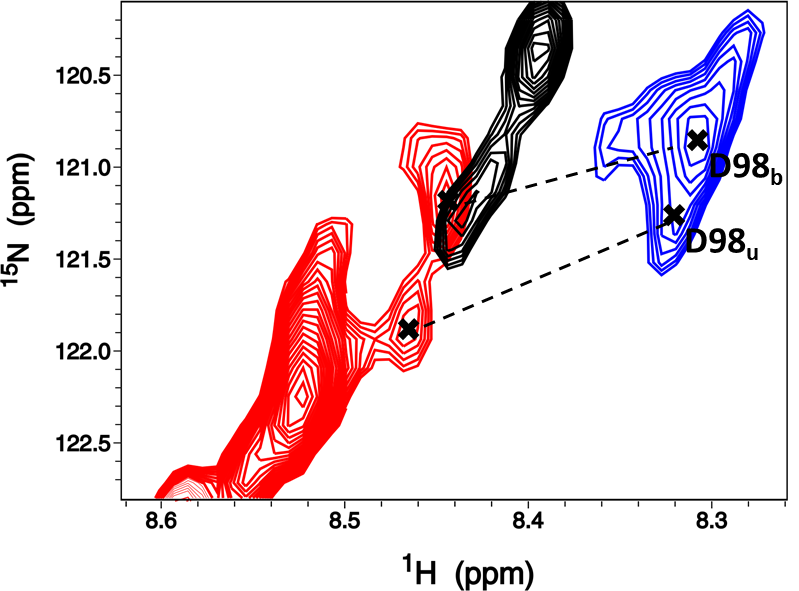


**Figure S11.** Overlapping of ^15^N-HSQC signals of D98 of αS-CEL in the absence (*black signal*) and in the presence of SDS micelles (*coloured signals*) at 12.5ºC (*black and red*) and at 37ºC (*blue*). Dashed lines represent the temperature-dependent shifting of the resonances. The signal corresponding to the unbound residue is labelled as “D98_u_”, whereas that corresponding to the SDS-bound forms is labelled as “D98_b_”.


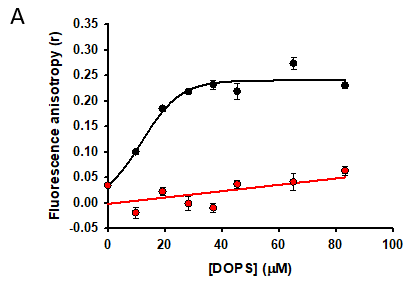


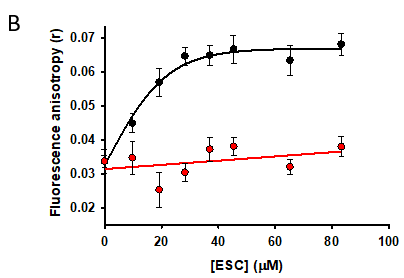


**Figure S12.** Studying the fluorescence anisotropies of αS and αS-CEL in the presence of SUVs. **(A)** Fluorescence anisotropy (*r*) of solutions containing αS (13 µM; *black*) or αS-CEL (13 µM; *red*) at different DOPS-SUVs concentrations. **(B)** Fluorescence anisotropy (*r*) of solutions containing αS (13 µM; *black*) or αS-CEL (13 µM; *red*) at different ESC-SUVs concentrations. All the measurements shown in panels **A** and **B** were carried out in 20 mM phosphate buffer (pH 7.4) containing 150 mM NaCl and at 25 °C. The continuous lines represent the theoretical function describing the fluorescence anisotropy change for αS and αS-CEL, which were obtained using Sigma Plot software.


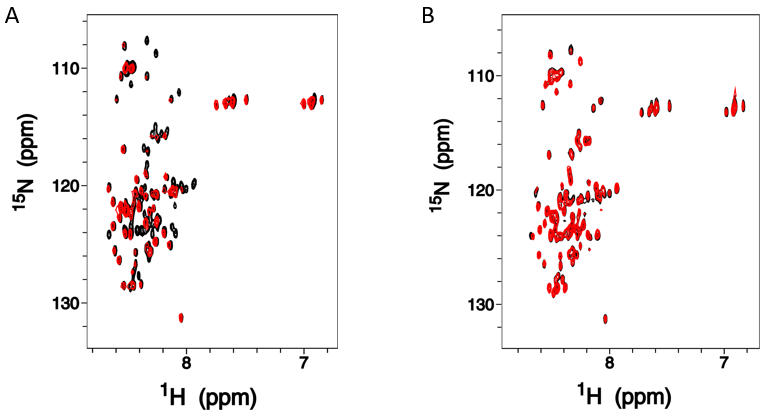


**Figure S13.** Overlapping of the ^1^H,^15^N-HSQC spectra obtained from solutions containing 135 µM αS (**A**) or αS-CEL (**B**) in the absence (*black*) and in the presence (*red*) of 1.3 mM ESC-SUVs. Experiments were acquired at 12.5 ºC in 20 mM phosphate buffer (pH 6.5).


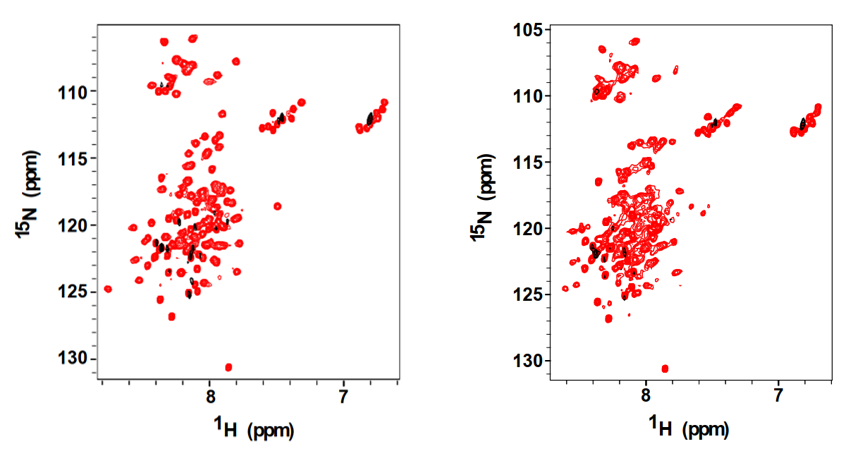


**Figure S14.** Effect of the presence of SDS micelles on the intensity (*I*) of the HSQC signals of αS and αS-CEL. **(A)** Overlapping of the ^15^N-HSQC spectra of αS before (*black*) and after the addition of 40 mM d_25_-SDS (*red*). For visualization purposes, the intensity of the HSQC spectrum collected in the absence of SDS (*black*) is twice than that of the HSQC spectrum collected in the presence of SDS (*red*). **(B)** Overlapping of the ^15^N-HSQC spectra of αS-CEL before (*black*) and after the addition of 40 mM d_25_-SDS (*red*). All the experiments were acquired at 37 ºC in 20 mM phosphate buffer (pH 6.5). For visualization purposes, the intensity of the HSQC spectrum collected in the absence of SDS (*black*) is eight times higher than that of the HSQC spectrum collected in the presence of SDS (*red*).


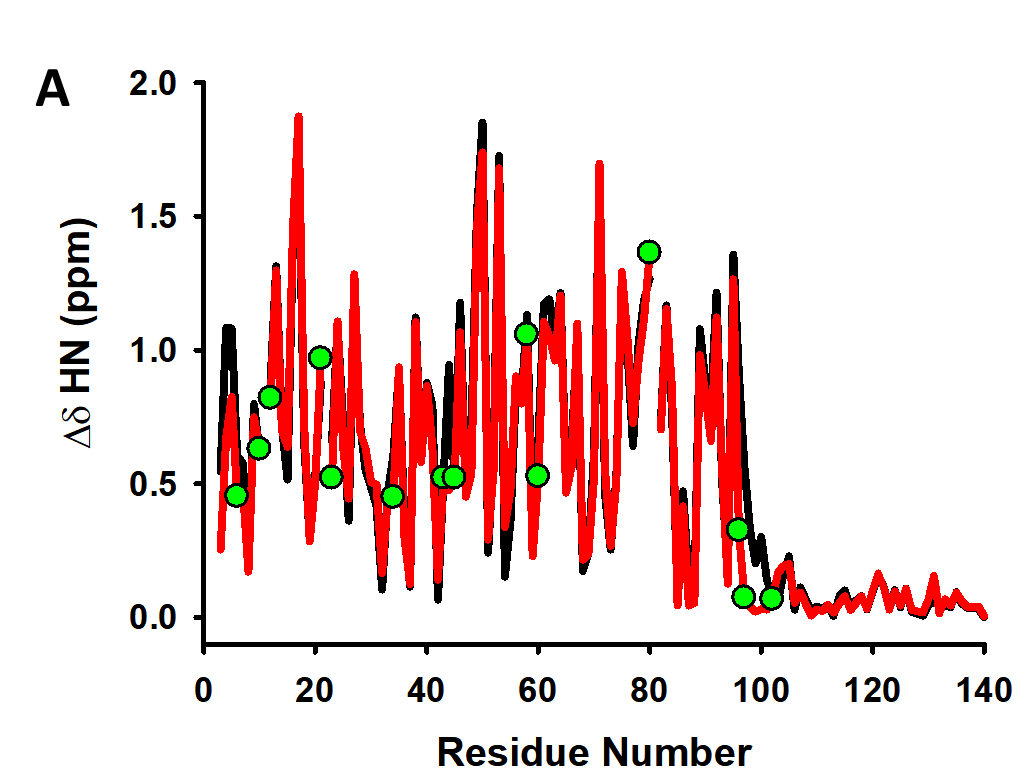


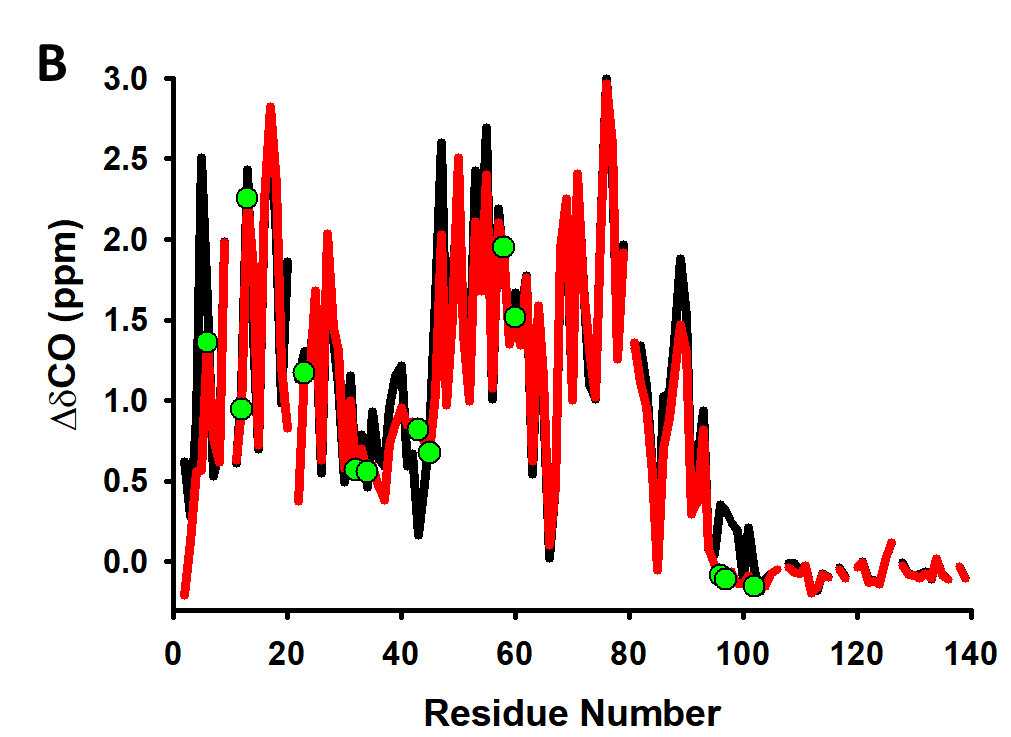


**Figure S15.** Effect of CEL on the chemical shifts of micelle bound αS. **(A)** Amide chemical shift perturbations (Δδ) of the HN and N backbone resonances of αS (*blac*k) and αS-CEL (*red*) as a result of their interaction to SDS micelles. Chemical shift assignments of HN and N resonances of the unbound states of αS and αS-CEL were achieved in a previous work of our group [10]. Here we have temperature-corrected those values and we have obtained the HN and N chemical shifts of their SDS-micelle bound states. For each residue, $\Delta\delta=\sqrt{\left( {\Delta\delta}_{HN} \right)^{2}+{x\cdot\left( {\Delta\delta}_{N} \right)}^{2}}$, where *x* is 0.2 for Gly and 0.14 for the other residues. Δδ_HN_ and Δδ_N_ are the amide proton and the amide nitrogen chemical shift differences, respectively. The positions of the CEL moieties along the sequence of αS-CEL are shown as *green* dots. **(B)** Chemical shift perturbations of the CO resonances (${\Delta\delta}_{CO}$) of αS (*black*) and αS-CEL (*red*) in function of the sequence as a result of their interactions with SDS micelles. Chemical shift assignments of the CO resonances of the unbound states of αS and αS-CEL were achieved in a previous work of our group [10]. Here we have temperature-corrected those values and we have obtained the CO chemical shifts of their SDS-micelle bound states. The positions of the CEL moieties along the sequence of αS-CEL are shown as *green* dots.


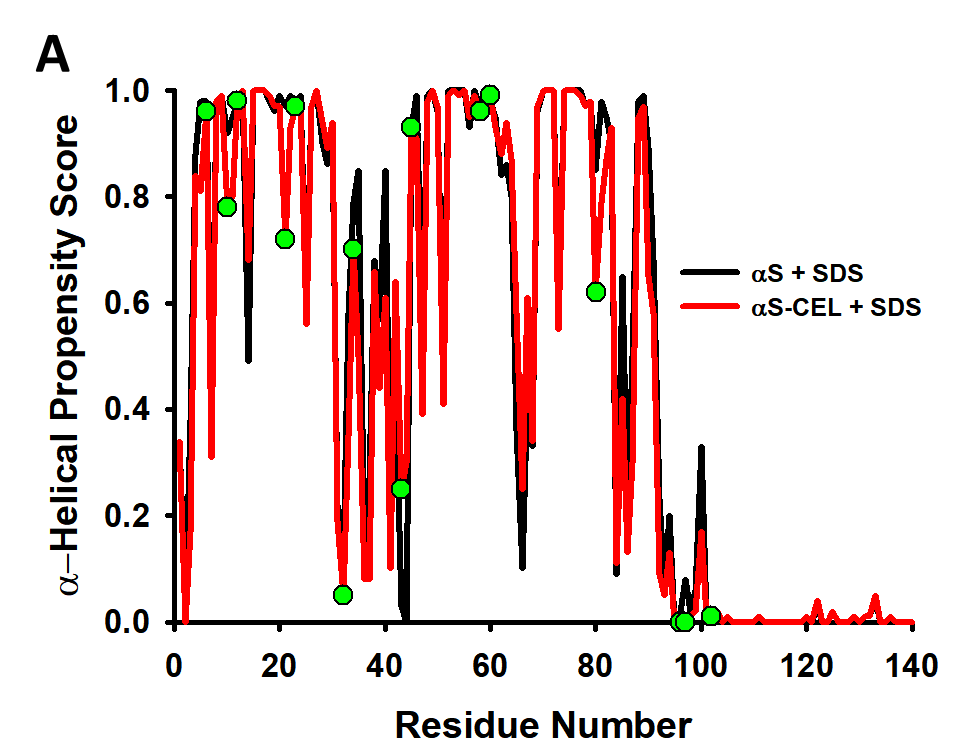


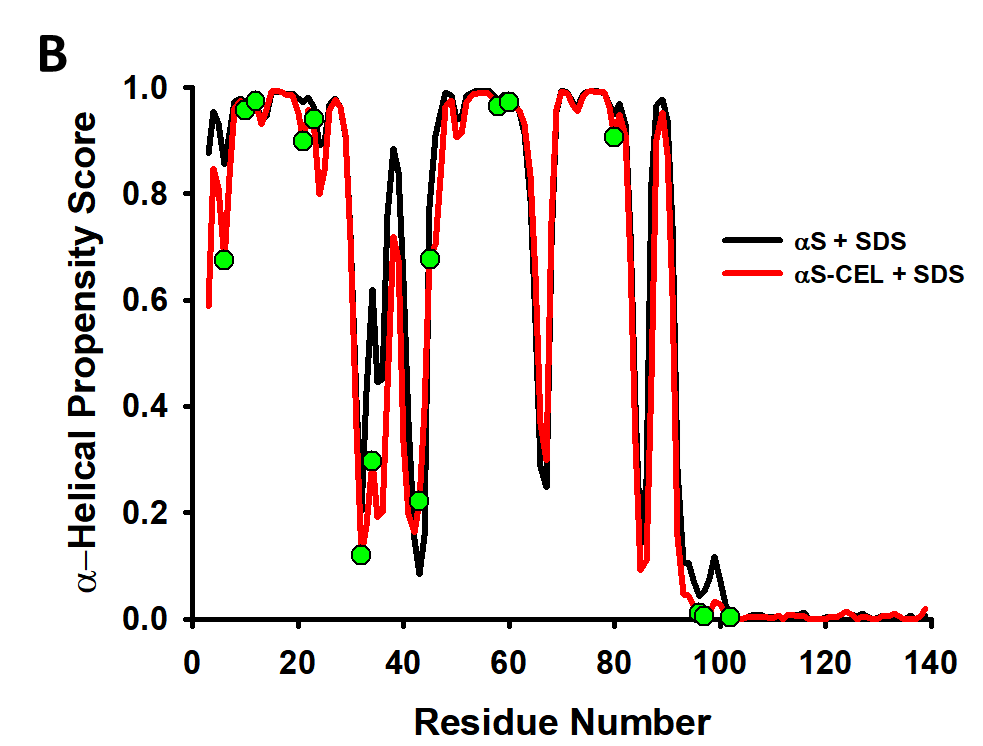


**Figure S16.** α-Helical propensity scores obtained for αS and αS-CEL. **(A)** α-Helical propensity scores obtained for αS (*black*) and αS-CEL (*red*) in the presence of SDS at 37 ºC and at pH 6.5. The values were obtained using the CSI3.0 web server (<http://csi3.wishartlab.com/cgi-bin/index.php>) and the corresponding HN, N, Hα, Cα, Cβ, and CO chemical shifts. **(B)** α-Helical propensity scores obtained for αS (*black*) and αS-CEL (*red*) in the presence of SDS at 37 ºC and at pH 6.5. The values were obtained using the Talos+ web server (https://spin.niddk.nih.gov/bax/nmrserver/talos/) and the corresponding HN, N, Hα, Cα, Cβ, and CO chemical shifts. In The positions of the CEL moieties along the sequence of αS-CEL are shown as *green* dots.


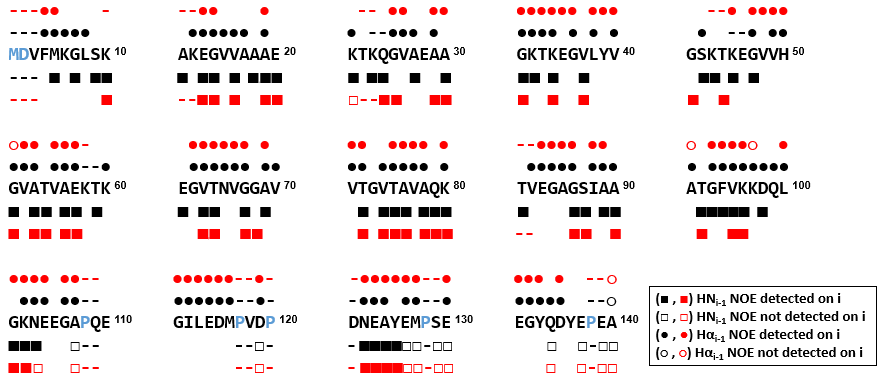


**Figure S17.** Primary sequence of αS complemented with NOE patterns observed for αS (*black*) and αS-CEL (*red*) in the presence of SDS, which are displayed above (Hα_i-1_/HN_i_; circles) and below (HN_i-1_/HN_i_; squares) the sequence. The NOE intensities for each residue were obtained from the corresponding ^1^H,^15^N-NOESY spectra. Residues coloured in blue were not included in the analysis of the sequential NOEs. Filled symbols display the residues for which the *i-1* NOE have been detected; empty symbols represent those residues for which *i-1* NOE was not observed; the absence of a symbol indicates that it was not possible to determine whether it was a NOE or not since the signal of the *i-1* residue overlapped with that of the *i* residue; and the script (-) was used for those residues that could not be assigned in the ^1^H,^15^N -NOESY spectra.


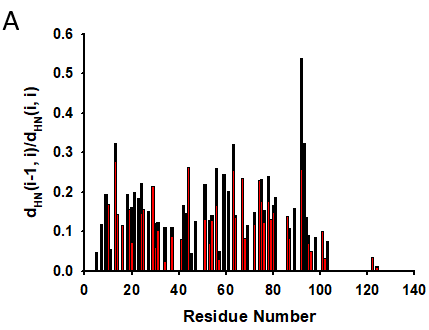


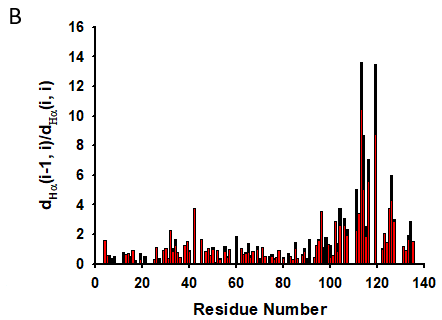


**Figure S18.** Sequential NOEs intensity ratios for αS (*black*) and αS-CEL (*red*) in the presence of SDS micelles. **(A)** Ratios of intraresidue to sequential H_N_-H_N_ NOE intensities in αS and αS-CEL. For comparison purposes, the plots only display the values for those residues whose *d*_NN_(*i,i*)/*d*_NN_(*i-1,i*) values could be determined for αS and αS-CEL. **(B)** Ratios of intraresidue to sequential H_α_-H_N_ NOE intensities in αS and αS-CEL. The NOE intensities corresponding to Gly residues were divided by 2, to correct the presence of two H_α_ atoms. For comparison purposes, the plots only display the values for those residues whose *d*_αN_(*i,i*)/*d*_αN_(*i-1,i*) values could be determined for αS and for αS-CEL.


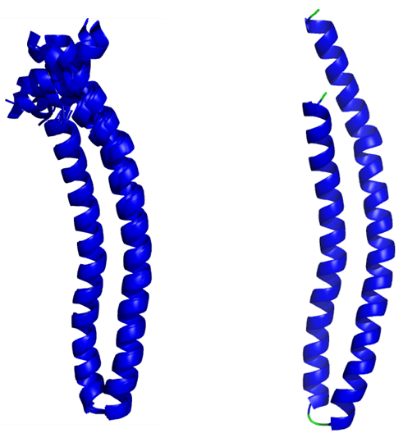

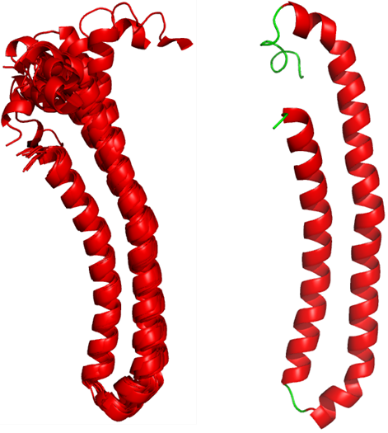


A

B

**Figure S19.** NMR structures of the micelle-bound αS and αS-CEL. **(A)** NMR bundle of the 10 lowest energy structures of αS. Blue cartoons connect the different backbone atoms *(left)*. The structures were aligned onto the V3-K43 stretch of the lowest energy structure. The average structure obtained from the ensemble using Molmol *(right)*. The disordered regions are colored in green. **(B)** NMR bundle of the 10 lowest energy structures of αS-CEL. Red cartoons connect the different backbone atoms *(left)*. The structures were aligned onto the V3-K43 stretch of the lowest energy structure. The average structure was obtained from the ensemble using Molmol *(right)*. The disordered regions are colored in green. The C-terminal region (G101-A140) of αS and αS-CEL lacks a well-defined secondary structure. Therefore, it is not shown in the structures of the panels **A** and **B** to have a better view of the structured regions.


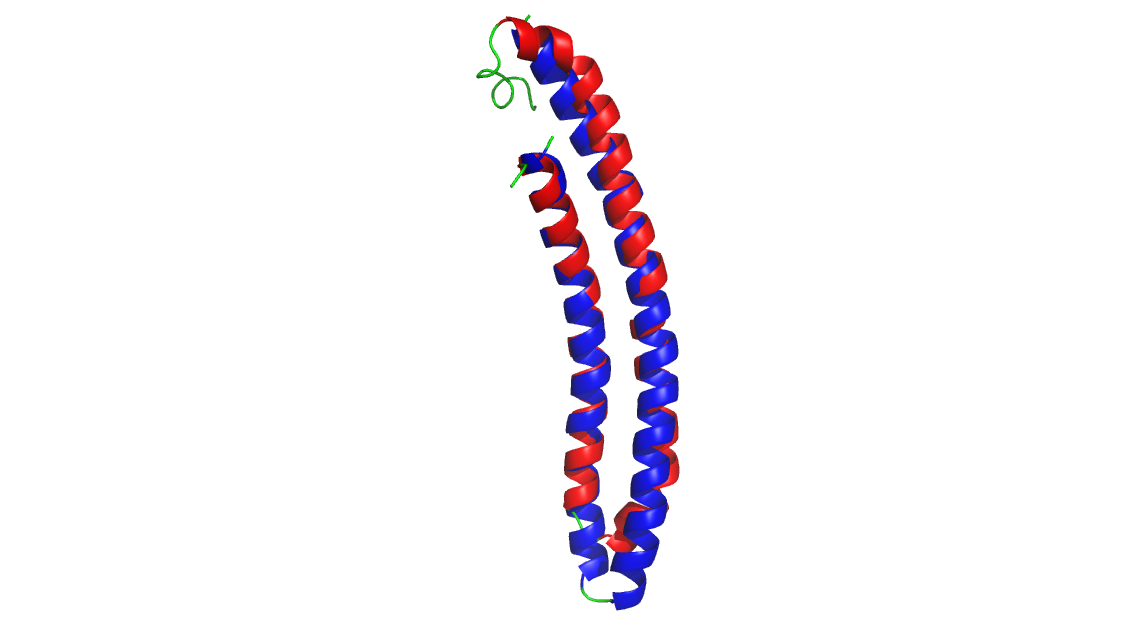


**Figure S20.** Overlapping of the average structures of SDS-bound αS (*blue*) and αS-CEL (*red*). The NMR solution structures were calculated using with PONDEROSA-software*.* The overlapping was carried out aligning the V3-E35 region using the Pymol software (RMDS 1.087Å). The disordered regions are colored in green.


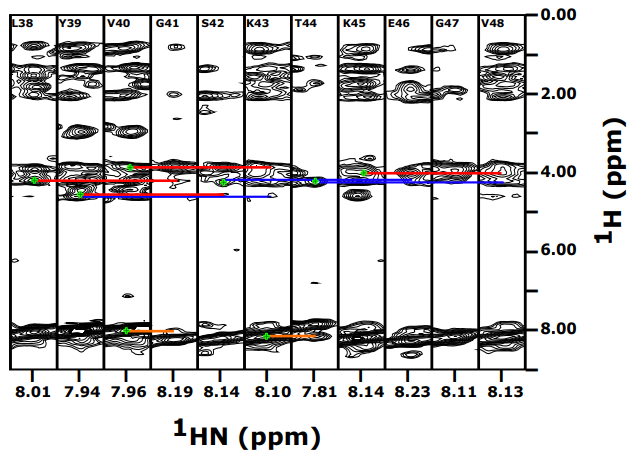


**Figure S21.** Strips of the three-dimensional ^1^H-^15^N-NOESY-HSQC spectrum of SDS-bound αS-CEL illustrating the NOE patterns observed for residues L38-V48. Solid *gold lines* appearing at ~8ppm (*y*-axis) connect the diagonal peaks with dNN(*i,i*+1) NOEs. Solid *red lines* appearing at ~4ppm (*y*-axis) connect intraresidue dαN(*i,i*) NOEs with dαN(*i,i*+3) connectivities. Solid *blue lines* appearing at ~4ppm (*y*-axis) connect intraresidue dαN(*i,i*) NOEs with dαN(*i,i*+4) connectivities.


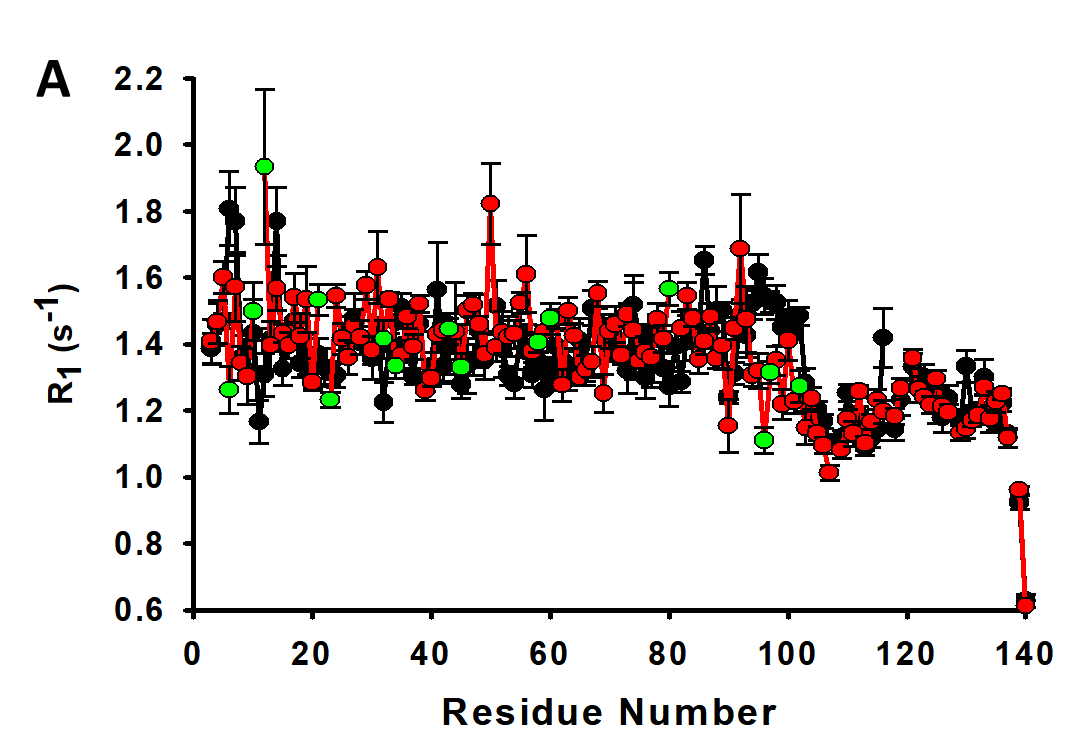


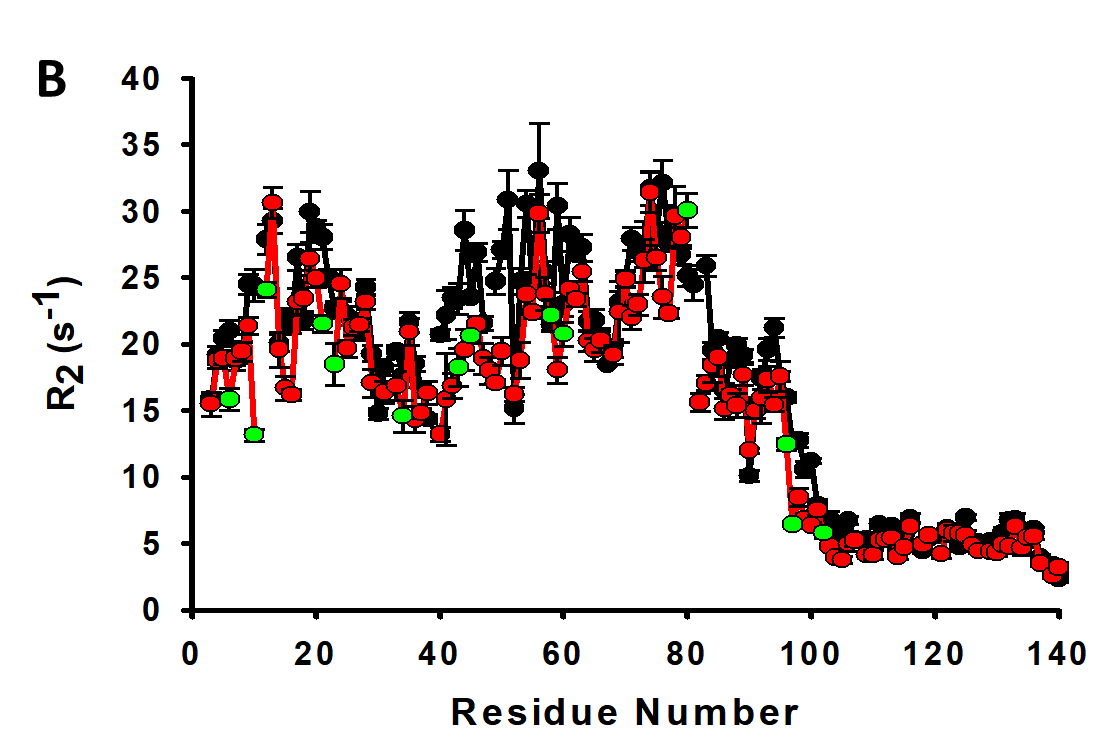


**Figure S22.** Effect of CEL formation on the dynamics of SDS-bound αS. **(A, B)** Plots of the R_1_ (s^-1^) (**A**) and R_2_ (s^-1^) (**B**) relaxation data obtained for αS (*black*) and αS-CEL (*red*) in the presence of SDS micelles. The experimental data corresponding to the different CEL moieties in αS-CEL is colored in *green*. The relaxation measurements were carried out at 37 ºC in 20 mM phosphate buffer (pH 6.5).

A B C

ESC/DPH

DOPC/DPH

DOPC/TMA-DPH

**Figure S23.** Lipid order parameters (S) of DOPC-based SUVs (130 µM) **(A, B)**, and ESC-based SUVs (130 µM) **(C)** in the absence (*black*) and in the presence of αS (*blue*) and αS-CEL (*red*). SUVs were labelled with TMA-DPH (2 µM) **(A)** and DPH (1 µM) **(B, C)** probes. Empty bars correspond to samples before the protein addition. Full bars represent the S values of the different SUVs after the addition of αS or αS-CEL (13 µM). Measurements were carried out in 20 mM phosphate buffer (pH 7.4) containing 150 mM NaCl at 25 ⁰C.

A B C

**Figure S24.** Calcein fluorescence intensity at 515 nm (λ_ex_ 495 nm) for solutions containing 130 µM DOPC **(A)**, DOPS **(B)** and ESC **(C)** SUVs in the absence (*grey*) and in the presence of 13 µM αS (*blue*), 13 µM αS-CEL (*red*), 26 µM αS (*green*) or 2 mM Triton X-100 detergent (used as control; *black*). All the experiments were carried out in 20 mM phosphate buffer (pH 7.4) containing 150 mM NaCl at 25 ⁰C.


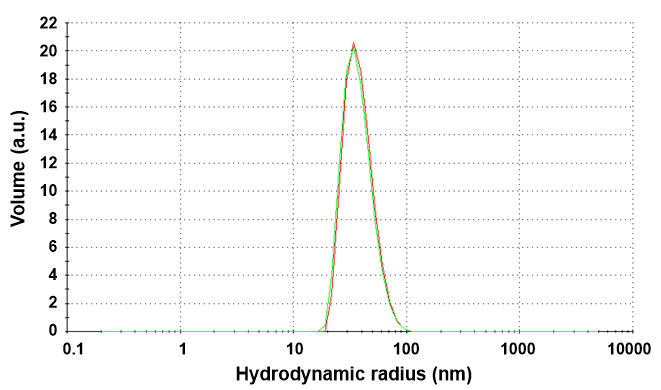


A

B

C


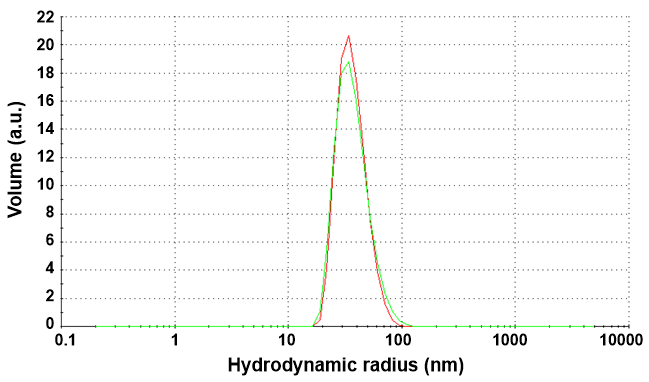


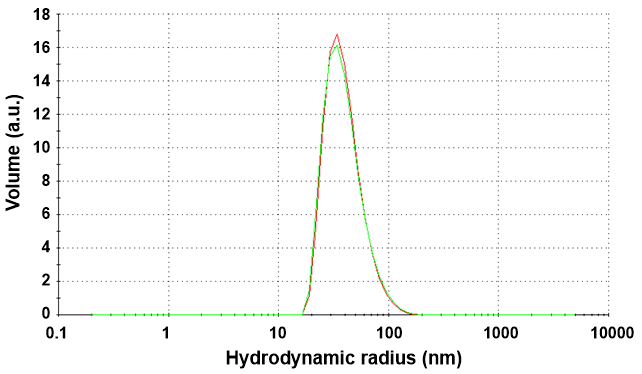


**Figure S25.** DLS size distributions of solutions containing 130 µM DOPS **(A)**, ESC **(B)** and DOPC **(C)** SUVs before (*red*) and after (*green*) 96 h of incubation. Measurements were performed in a 20 mM phosphate buffer (pH 7.4) containing 150 mM NaCl and at 25 ⁰C.


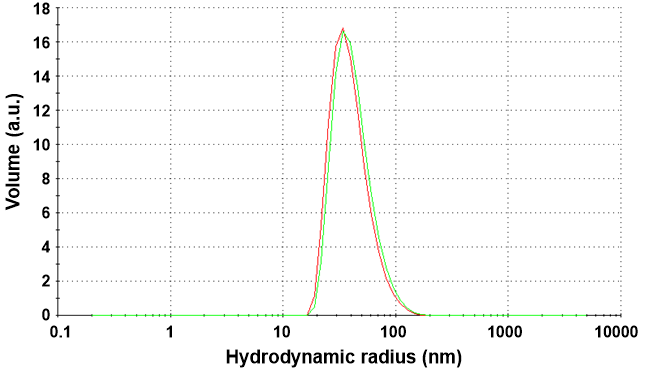

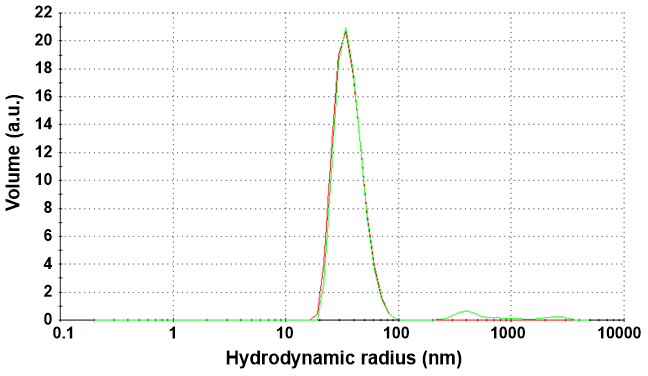


A B

C D


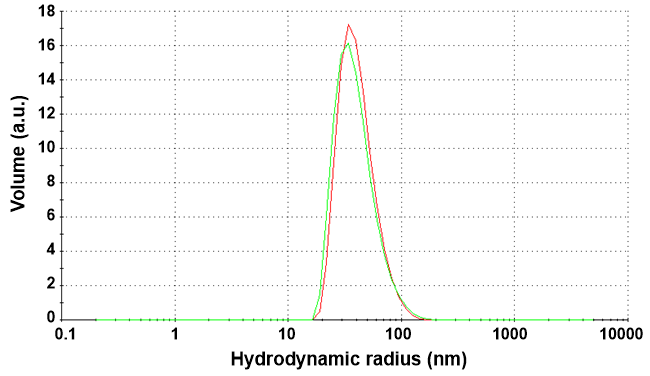

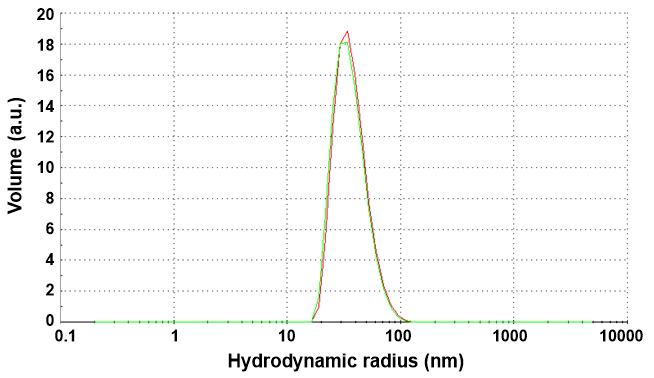


**Figure S26.** DLS size distributions of solutions containing 130 µM DOPC **(A, C)** or ESC **(B, D)** SUVs before (*red*) and after (*green*) 96 h of incubation in the presence of 13 µM αS **(A, B)** or αS-CEL **(C, D)**. Measurements were carried out in 20 mM phosphate buffer (pH 7.4) containing 150 mM NaCl at 25 ⁰C.


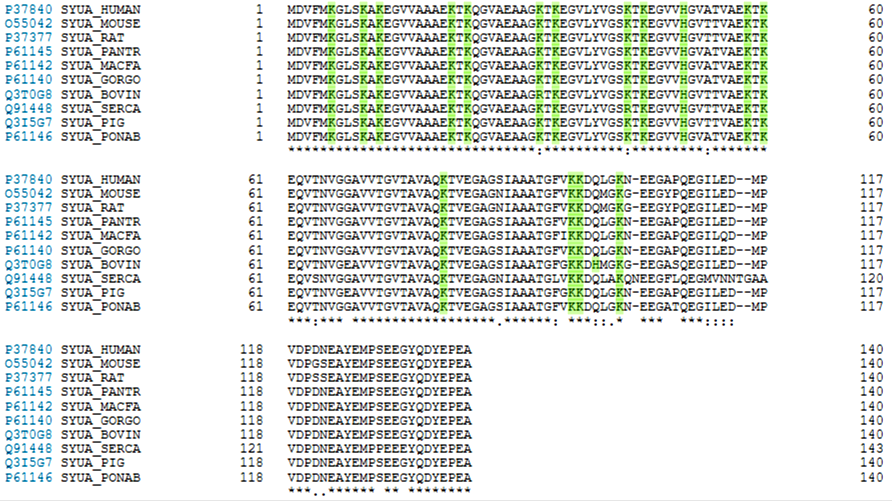


**Figure S27.** Sequence alignment and relationships of ten different αS sequences obtained from the Uniprot data base (https://www.uniprot.org/). The ten different sequences were aligned using the facility provided by the same database. The cationic residues (Lys, Arg and His) are highlighted in green.


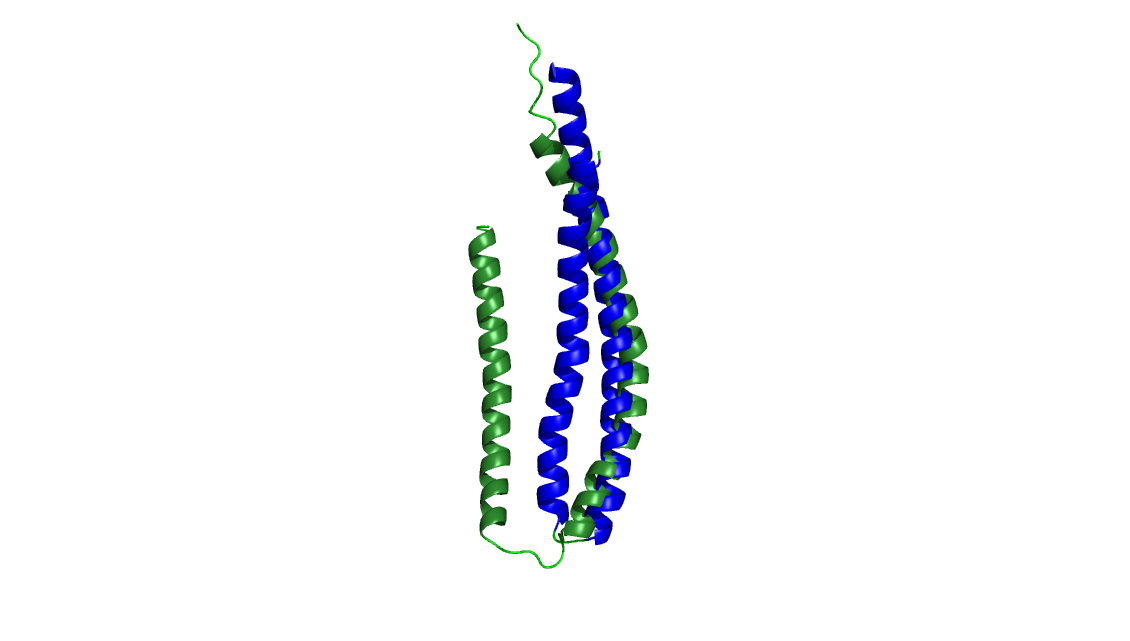

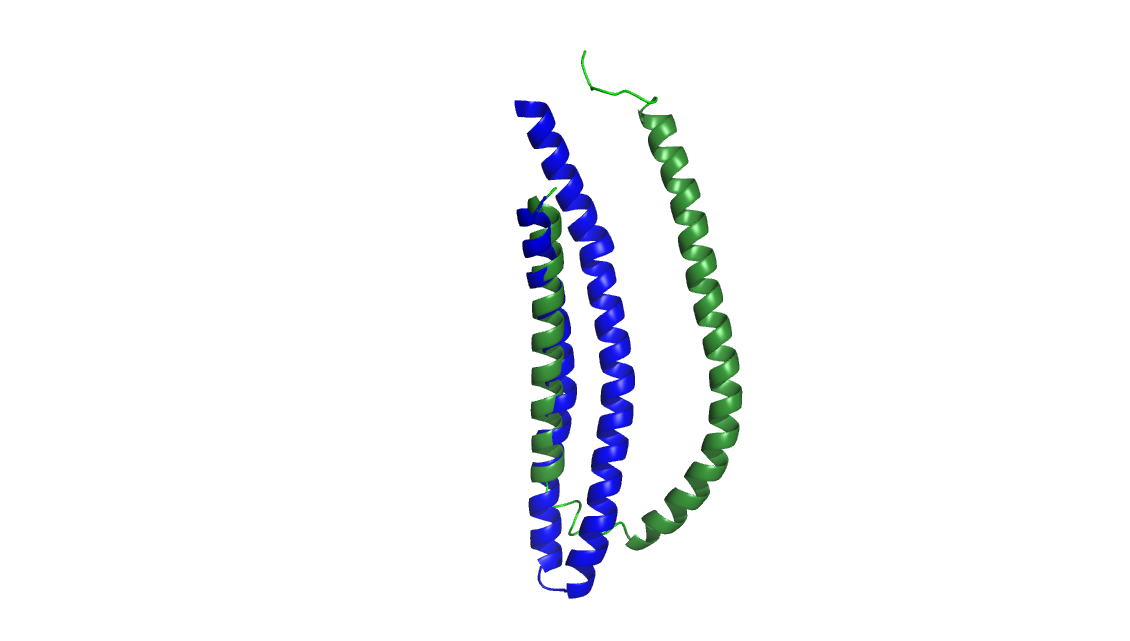
 .

A B

**Figure S28.** NMR structures of the micelle-bound αS. **(A)** Structural alignment of the K45-T92 region corresponding to the published NMR structure of the SDS-bound αS (PDB: 1XQ8) [2] *(green),* onto the same stretch of the average NMR structure of the SDS-bound αS calculated in this study (*blue*) (RMDS 3.274Å). **(B)** Structural alignment of the D2-K32 region corresponding to the first model of the NMR structural ensemble of αS obtained in the presence of sodium lauroyl sarcosinate (PDB: 2KKW) [3] *(green),* onto the same stretch of the average NMR structure of the SDS-bound αS calculated in this study (*blue*) (RMDS 2.055Å). The disordered regions are colored in green. The C-terminal region of the obtained structures (G101-A140) lacks a well-defined secondary structure, and it is not shown in the structures to have a better view of the structured regions.


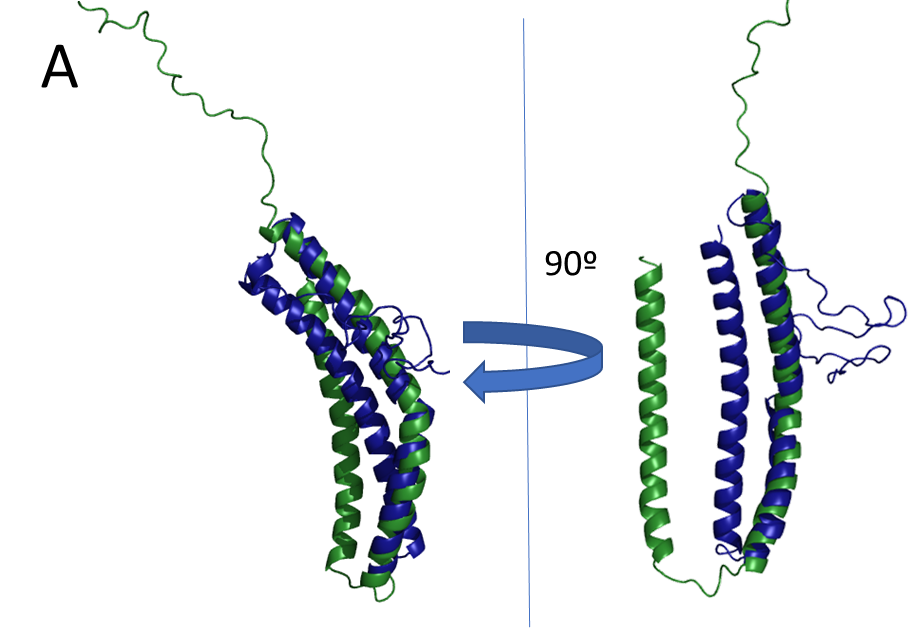


A

B

C


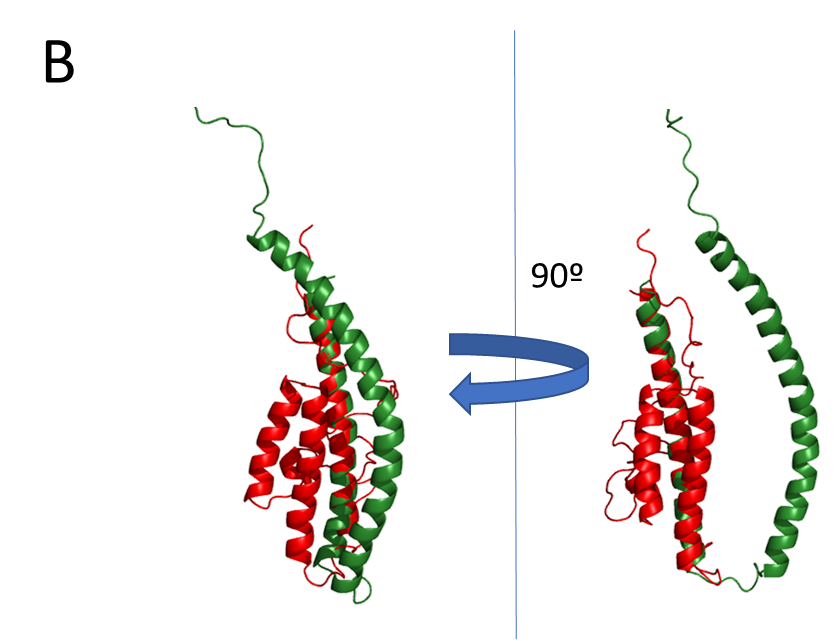
 ,


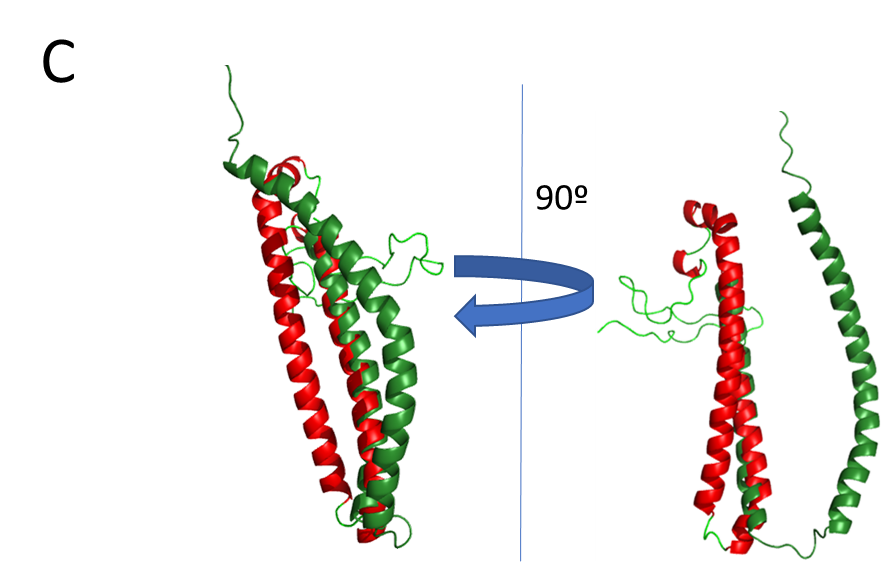
,

**Figure S29.** Using CS-Rosetta to model the SDS-micelle bound structures of αS and αS-CEL. **(A)** Structural alignment of the K45-T92 region in the published NMR structure of SDS-bound αS (PDB: 1XQ8) [2] *(green)*, onto the same stretch of the lowest energy model of the SDS-bound form of αS obtained using CS-Rosetta (*blue*). **(B)** Structural alignment of the V3-G36 region in the published NMR structure of SDS-bound αS (PDB: 1XQ8) [2] *(green)*, onto the same stretch of the lowest energy model of SDS-bound form of αS-CEL obtained using CS-Rosetta (*red*). **(C)** Structural alignment of the V3-G36 region in the published NMR structure of SDS-bound αS (PDB: 1XQ8) [2] *(green),* onto the same stretch of the SDS-bound structure of αS-CEL that had the second lowest energy according to CS-Rosetta (*red*). In all panels, two different views are shown with a 90° rotation.

A

B


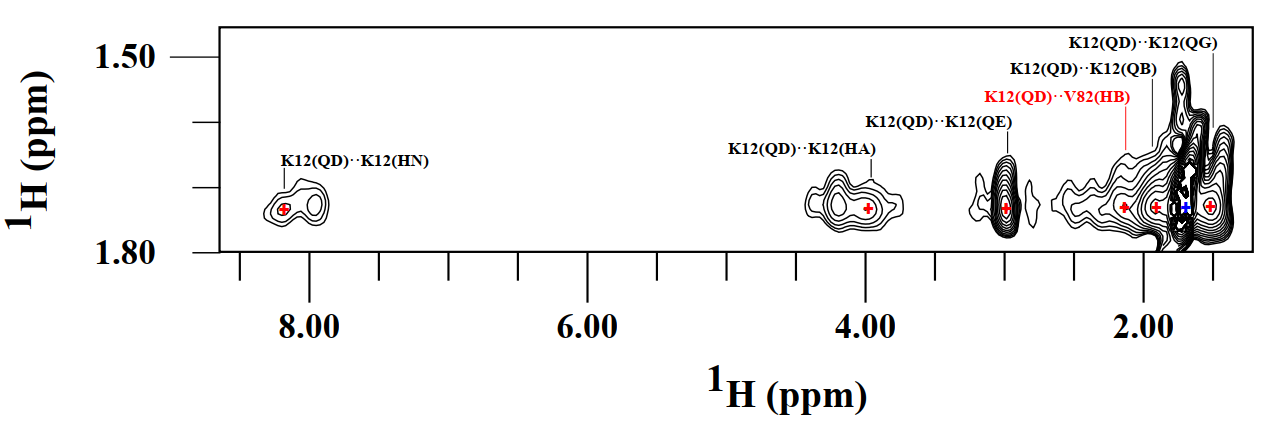


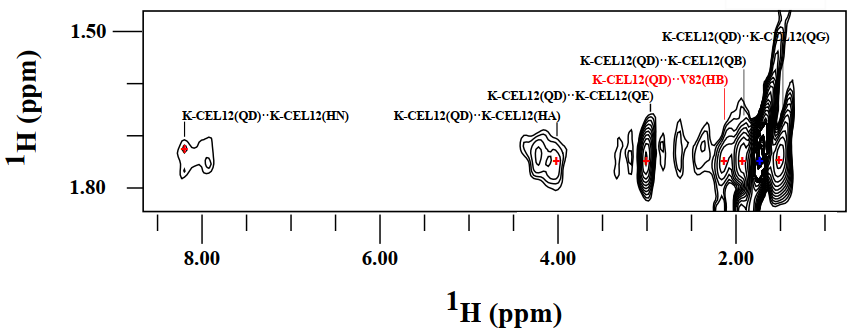


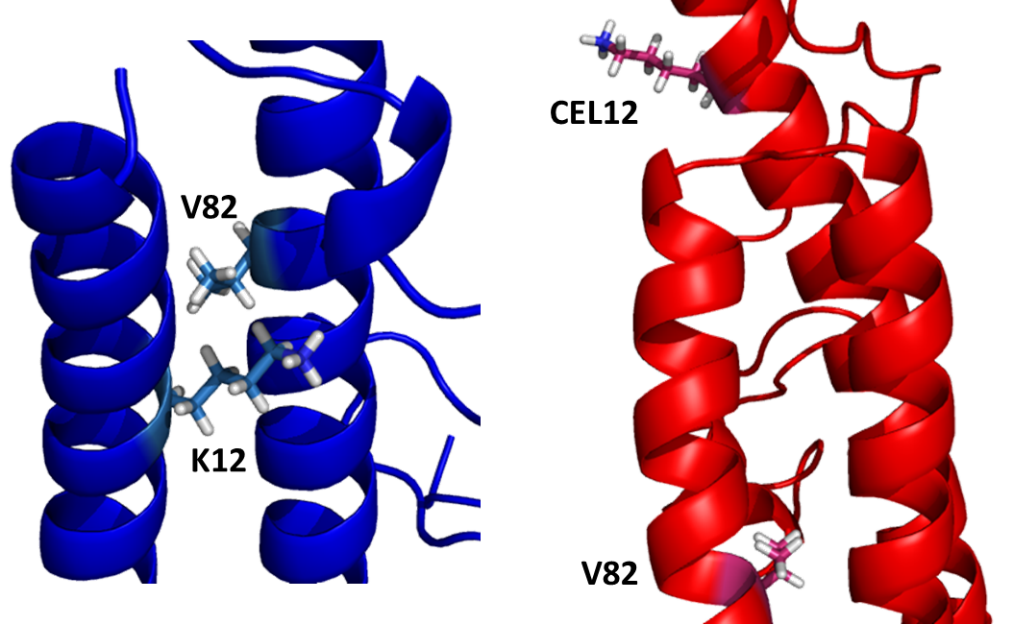


**Figure S30.** Using ^13^C-NOESY-HSQC NOEs to select the best CS-Rosetta model for the structure determination of micelle-bound αS-CEL. **(A)** ^13^C-NOESY-HSQC planes corresponding to the NMR frequencies of C_δ_ in K12 (*top;* 29.738 ppm) or in CEL12 (*bottom;* 28.986 ppm) of the micelle-bound αS (*top*) and αS-CEL *(bottom)*. NOE signals are labelled with a cross. Blue crosses correspond to the H_δ_-H_δ_ cross-peaks of K12 in αS *(top)* and of CEL12 in αS-CEL *(bottom).* Intraresidual contacts are labelled in *black*, whereas interresidual contacts are labelled in *red*. **(B)** Zoom on the C-terminal and N-terminal contact region of the lowest energy CS-Rosetta models of the SDS-bound αS (*blue; left*) and αS-CEL model (*right;* *red*). The side chains of K12/CEL12 and V82 are labelled and shown as sticks.

**Supplementary Tables**

**Table S1.** SAXS-derived structural dimensions of the SUVs used in this study. The dimensions were obtained from the fitting of the experimental scattering curves to the *lamellar_slab_APL_nW* model implemented in the SasView suite.

| Structural Parameters | ESC-SUVs | DOPS-SUVs | DOPC-SUVs |
| --- | --- | --- | --- |
| Thickness of the bilayer (Å) | 32.3±0.3 | 33.2±0.2 | 23.8±1 |
| Thickness of the head group (Å) | 4.3±0.2 | 4.7±0.1 | 4.0±0.8 |
| Thickness of the hydrophobic core of the bilayer (Å) | 23.5±0.4 | 23.8±0.2 | 15.8±2 |

**Table S2.** Structural statistics for the calculations of the αS and αS-CEL structures in the presence of SDS. Statistics were calculated for the 10 lowest energy structures after water refinement.

|  | αS | αS-CEL |
| --- | --- | --- |
| Final NMR restrains | **1007** | **936** |
| Short-range (i-j) ≤ 1 | 731 | 695 |
| Medium-range 1 < (i-j) < 5 | 236 | 195 |
| Long-range (i-j) ≥ 5 | 40 | 46 |
| Dihedral angle (Φ) constraints^a^ | 137 | 136 |
| Dihedral angle (Ψ) constraints^a^ | 136 | 132 |
|  |  |  |
| Restraints statistics^b^ |  |  |
| Distance violations > 0.0 Å | 0 | 0 |
| Torsion angle violations > 0º | 0 | 0 |
|  |  |  |
| Pairwise RMSD of residues V3-K43 in Å^c^ |  |  |
| Backbone N, Ca, CO | 0.53±0.20 | 0.54±0.18 |
| Heavy atoms | 1.23±0.22 | 1.27±0.24 |
|  |  |  |
| Ramachandran plot^d^ |  |  |
| Most favoured regions (%) | 98.1 | 98.5 |
| Additional allowed regions (%) | 1.9 | 1.5 |
| Generously allowed regions (%) | 0.0 | 0.0 |
| Disallowed regions (%) | 0.0 | 0.0 |
| ^a^Derived from Preditor.  ^b^Violations are only reported when present in five or more structures.  ^c^Coordinate precision is given as the average pair-wise Cartesian coordinate root mean square deviations over the ensemble.  ^d^Values obtained from the PROCHECK-NMR analysis [11] by using the Protein Structure Validation Server (PSVS) [12] | | |

**Table S3.** Hydrodynamic radius data (nm) from DLS size measurements of a solution containing 130 µM DOPS-, ESC- and DOPC-SUVs incubated during 96 h in the absence (control) or in the presence of 13 µM αS or αS-CEL.

| SUVs composition | DOPC | DOPS | ESC |
| --- | --- | --- | --- |
| Control | 52.85±25.17 | 45.64±14.17 | 45.64±16.84 |
| αS | 52.85±24.07 | 39.41±8.65 (74%)  307.6±91.5 (22.2%)  2780±165.9 (3.3%) | 39.41±12.62 (95.8%)  553.2±339.5 (3.3%)  2780±367.8 (0.9%) |
| αS-CEL | 52.85±22.03 | 45.64±12.81 | 45.64±16.20 |

**REFERENCES**

1. Lange OF, Rossi P, Sgourakis NG, Song Y, Lee HW, Aramini JM, Ertekin A, Xiao R, Acton

TB, Montelione GT, Baker D (2012) Determination of solution structures of proteins up to

40 kDa using CS-Rosetta with sparse NMR data from deuterated samples. Proc Natl Acad

Sci USA 109:10873-8.

2. Ulmer TS, Bax A, Cole NB, Nussbaum RL (2005) Structure and dynamics of micelle-bound

human alpha-synuclein. J Biol Chem 280:9595-603.

3. Rao JN, Jao CC, Hegde BG, Lange R, Ulmer TS (2010) A combinatorial NMR and EPR

approach for evaluating the structural ensemble of partially folded proteins. J Am Chem Soc

132:8657-68.

4. Allison JR, Varnai P, Dobson CM, Vendruscolo M (2009) Determination of the free energy landscape of alpha-synuclein using spin label nuclear magnetic resonance measurements. J Am Chem Soc 131:18314-18326.

5. Frishman D, Argos P (1995) Knowledge-based protein secondary structure assignment. Proteins 23:566-579.

6. Ulrich P, Cerami A (2001) Protein glycation, diabetes, and aging. Recent Prog Horm Res 56:1–22.

7. Allaman I, Bélanger M, Magistretti PJ (2015) Methylglyoxal, the dark side of glycolysis. Neurosci 9:23.

8. Vicente Miranda H, Szego ÉM, Oliveira LMA, Breda C, Darendelioglu E, de Oliveira RM, Ferreira DG, Gomes MA, Rott R, Oliveira M, Munari F, Enguita FJ, Simões T, Rodrigues EF, Heinrich M, Martins IC, Zamolo I, Riess O, Cordeiro C, Ponces-Freire A, Lashuel HA, Santos NC, Lopes LV, Xiang W, Jovin TM, Penque D, Engelender S, Zweckstetter M, Klucken J, Giorgini F, Quintas A, Outeiro TF (2017) Glycation potentiates α-synuclein-associated neurodegeneration in synucleinopathies. Brain 140:1399-1419.

9. Tan L, Elkins J G, Davison B H, Kelley E G, Nickels J (2021) Implementation of a self-consistent slab model of bilayer structure in the SasView suite. J Appl Cryst 54:363-370.

10. Mariño L, Ramis R, Casasnovas R, Ortega-Castro J, Vilanova B, Frau J, Adrover M (2020) Unravelling the effect of N(ε)-(carboxyethyl)lysine on the conformation, dynamics and aggregation propensity of α-synuclein. Chem Sci 11:3332-3344.

11. Laskowski RA, Rullmann JA, MacArthur MW, Kaptein R, Thornton JM (1996) AQUA and PROCHECK-NMR: Programs for checking the quality of protein structures solved by NMR. J Biomol NMR 8:447.

12. Bhattacharya A, Tejero R, Montelione GT (2007) Evaluating protein structures determined by structural genomics consortia. Proteins 66:778.
